# Exon-variant interplay and multi-modal evidence identify endocrine dysregulation in severe psychiatric disorders impacting excitatory neurons

**DOI:** 10.1038/s41398-025-03366-8

**Published:** 2025-04-19

**Authors:** Karolina Worf, Natalie Matosin, Nathalie Gerstner, Anna S. Fröhlich, Anna C. Koller, Franziska Degenhardt, Holger Thiele, Marcella Rietschel, Madhara Udawela, Elizabeth Scarr, Brian Dean, Fabian J. Theis, Nikola S. Mueller, Janine Knauer-Arloth

**Affiliations:** 1 Institute of Computational Biology, Helmholtz Center, Munich, Germany; 2 TUM School of Life Sciences Weihenstephan, Technical University of Munich, Freising, Germany; 3 Department of Gene and Environment, Max Planck Institute of Psychiatry, Munich, Germany; 4 School of Medical Sciences, Faculty of Medicine and Health, University of Sydney, Camperdown, NSW Australia; 5 Charles Perkins Centre, University of Sydney, Camperdown, NSW Australia; 6 International Max Planck Research School for Translational Psychiatry, Max Planck Institute of Psychiatry, Munich, Germany; 7 Institute of Human Genetics, University of Bonn, Bonn, Germany; 8 Department of Genomics, Life & Brain Center, University of Bonn, Bonn, Germany; 9 Cologne Center for Genomics, University of Cologne, Cologne, Germany; 10 Department of Genetic Epidemiology in Psychiatry, Central Institute of Mental Health, University Medical Center Mannheim/University of Heidelberg, Mannheim, Germany; 11 The Molecular Psychiatry Laboratory, The Florey Institute of Neuroscience and Mental Health, Parkville, VIC Australia; 12 The Department of Psychiatry, The University of Melbourne, Parkville, VIC Australia; 13 The Department of Florey, The University of Melbourne, Parkville, VIC Australia; 14 TUM School of Computation, Information and Technology, Technical University of Munich, Garching, Germany

**Keywords:** Molecular neuroscience, Personalized medicine, Medical genetics, Predictive markers, Psychiatric disorders

## Abstract

Bipolar disorder (BD), major depressive disorder (MDD), and schizophrenia share genetic architecture, yet their molecular mechanisms remain elusive. Both common and rare genetic variants contribute to neural dysfunction, impacting cognition and behavior. This study investigates the molecular effects of genetic variants on human cortical single-cell types using a single-exon analysis approach. Integrating exon-level eQTLs (common variants influencing exon expression) and joint exon eQT-Scores (combining polygenic risk scores with exon-level gene expression) from a postmortem psychiatric cohort (BD = 15, MDD = 24, schizophrenia = 68, controls = 62) with schizophrenia-focused rare variant data from the SCHEMA consortium, we identified 110 core genes enriched in pathways including circadian entrainment (FDR = 0.02), cortisol synthesis and secretion (FDR = 0.026), and dopaminergic synapse (FDR = 0.038). Additional enriched pathways included hormone signaling (FDRs < 0.0298, including insulin, GnRH, aldosterone, and growth hormone pathways) and, notably, adrenergic signaling in cardiomyocytes (FDR = 0.0028). These pathways highlight shared molecular mechanisms in the three disorders. Single-nuclei RNA sequencing data from three cortical regions revealed that these core set genes are predominantly expressed in excitatory neuron layers 2–6 of the dorsolateral prefrontal cortex, linking molecular changes to cell types involved in cognitive dysfunction. Our results demonstrate the power of integrating multimodal genetic and transcriptomic data at the exon level. This approach moves beyond symptom-based diagnoses toward molecular classifications, identifying potential therapeutic targets for psychiatric disorders.

## Introduction

Psychiatric disorders such as bipolar disorder (BD), major depressive disorder (MDD) and schizophrenia are characterized by shared pathophysiological, clinical and biological features, often involving alterations in neural circuits within the prefrontal cortex (PFC) [1]. These brain areas are critical for cognition and executive functioning, processes frequently affected in psychiatric disorders [2]. Understanding how these impairments manifest requires exploring the underlying biological mechanisms. Cross-disorder psychiatric studies provide a valuable framework for investigating these shared processes, moving beyond surface-level phenotypic features such as symptomatology and diagnostic criteria. By exploring commonalities across disorders, we gain deeper insights into the underlying mechanisms that contribute to psychiatric illness.

Postmortem brain specimens provide a unique opportunity to investigate disease-related changes at the cellular and molecular level and explore transcriptome-wide molecular processes in neural circuits, which is important given that this is the level at which novel treatment targets can be identified.

Genome-wide association studies (GWASs) are powerful tools for understanding genetic predisposition of complex psychiatric disorders and have successfully identified numerous disease-associated genetic loci. For example, Trubetskoy et al. conducted a GWAS on schizophrenia with 76 755 cases and 243 649 controls, identifying 287 genetic risk loci significantly enriched in gene expression of the Brodmann area 9 (BA9) PFC region [3]. Based on the single variant GWAS results the idea of calculating polygenic risk scores (PRSs) was developed [4]. The PRS quantifies the cumulative genetic risk for a disorder, yet its predictive diagnostic accuracy in psychiatry is still evolving, with limited explained variance (e.g., 7.3% for schizophrenia [3]). Improvements in statistical methods for PRS calculation and inclusion of larger and more diverse GWAS datasets hold promise for increasing this accuracy and refining our genetic understanding of psychiatric disorders as well as potentially enabling computer-aided diagnosis [5].

In addition to GWASs focusing on common single nucleotide polymorphisms (SNPs), investigations of rare, pathogenic variants may also substantially enhance our understanding of the genetic bias of psychiatric disorders. Wainschtein et al. [6] proposed that the lower heritability of common SNPs compared to population-based estimates is attributed to rare variants, which are often located in regions of low linkage disequilibrium and are more likely to be protein altering. This finding aligns with previous findings showing that rarer and evolutionarily younger SNPs exhibit greater SNP heritability in various complex traits, including BD, MDD and schizophrenia [7]. Notably, rare coding variants identified as major contributors to schizophrenia risk, with ten genes highlighted by Singh et al. [8], play a critical role in bridging molecular interruptions and disease phenotypes in psychiatric genetics. Furthermore, a study found evidence for an additive model in which the presence of rare copy number variants (CNVs) reduces the impact of common SNPs on schizophrenia risk, highlighting the interplay of common and rare variants [9]. Additionally, studies utilizing postmortem brain samples from established large cohorts, such as Genotype-Tissue Expression (GTEx) [10], CommonMind Consortium (CMC) [11] and PsychENCODE [12] have identified large-scale molecular changes in gene expression and regulation. Recent advancements in single-cell/nucleus RNA-sequencing (sc/snRNA-seq) technologies have built on these works to enable the study of cell-type-specific gene expression, as demonstrated recently in the first single-cell atlas for schizophrenia, which revealed differentially expressed genes in inhibitory and excitatory neurons [13].

GWASs often pinpoint significant variants in non-coding regions [14], many of which influence gene expression as expression quantitative trait loci (eQTLs) [15]. SNPs associated with schizophrenia are enriched for eQTLs [16, 17]. While subgene-level eQTL studies, such as those focusing on alternative splicing (splicingQTLs) [18], have demonstrated their importance in complex disorders like schizophrenia [19, 20], a comprehensive understanding requires investigating regulatory effects at the exon level. Exon-level analyses, as demonstrated by Jaffe et al. [21], can reveal transcript-specific gene regulation missed by traditional gene-level approaches. This study emphasizes the importance of accurately quantifying exon-level expression to identify subtle but potentially impactful genetic effects. However, there has been little previous exploration of genetic effects on exon-level expression in the context of psychopathology, using a focused approach for accurate quantification [22].

To identify these key regulatory elements and gain a deeper understanding of their role in psychiatric disorders, we hypothesized that integrating common variant effects with rare variant data would identify core disease mechanisms, specifically genes and pathways where both common and rare variation contribute to dysregulation. Common variants may exert subtle, widespread effects, while rare variants can be more disruptive; convergent findings across both types of variation highlight robust targets. Therefore, this study investigates genetic effects on exons and their correlation with affected cell types in the human PFC (BA9). Specifically, we aimed to identify genes and pathways showing convergent evidence of dysregulation across common and rare variant data and to determine the cell-type and layer-specific expression of these prioritized genes within the PFC (BA6, BA9, BA10, and BA11). This multi-faceted approach allowed us to explore the interplay of genetic risk, gene expression at the exon level, and cell-type localization in contributing to the pathophysiology of psychiatric disorders. Our findings contribute to a broader understanding of psychiatric disorder development and inform the identification of potential therapeutic targets and intervention approaches.

## Materials and methods

### Study samples, tissue collection and processing

The primary cohort (Dataset 1 - exon array data) has been previously described in Scarr et al. [23] and Dean et al. [24]. Briefly, dorsolateral prefrontal cortex (DLPFC) tissue from 169 adult subjects aged 18–87 years was included in the study. The cohort comprised individuals diagnosed with BD (n = 15), MDD (n = 24) and schizophrenia (n = 68) and 62 subjects with no psychiatric diagnosis (refer to Table S1). Demographic, clinical and pharmacological data were obtained during a case history review conducted using the Diagnostic Instrument for Brain Studies (DIBS), as described previously [23]. Tissue collection and processing was performed as described previously [23]. All tissue was obtained from the Victorian Brain Bank at the Florey Institute for Neuroscience and Mental Health. Brodmann area 9 (BA 9) was taken from the lateral surface of the frontal lobe from an area comprising the middle frontal superior gyrus to the inferior frontal sulcus of the left hemisphere.

Dataset 2 consisted of multiple human postmortem brain cohorts utilized for downstream single-nucleus RNA-sequencing (snRNA-seq) analysis. (a) Postmortem orbitofrontal cortex tissues from Brodmann area 11 (BA11) obtained from two neurotypical individuals (Table S1) were used for snRNA-seq. These brain tissues were fresh-frozen and obtained from the NSW Brain Tissue Resource Centre in Sydney, Australia. BA 11 was dissected from the 3rd 8–10 mm coronal slice from each fresh hemisphere for each subject, guided by visual inspection of neuroanatomical landmarks (primarily the straight and medial orbital gyri) in a slice anterior to the corpus callosum. This approach ensured consistent dissection across subjects. (b) Publicly available snRNA-seq data from BA9 postmortem brain tissue obtained from 17 individuals not diagnosed with a psychiatric disorder [25] were downloaded from the NCBI database under GEO accession number GSE144136. (c) Publicly available snRNA-seq data from BA6 and BA10 postmortem brain tissue obtained from six neurotypical adult human brains [26], GSE97930, were used in WebCSEA.

### Gene expression data

For Dataset 1, gene expression analysis was conducted using exon arrays. Total RNA was extracted from frozen gray matter, with RNA quantity and quality subsequently assessed. The expression arrays were processed utilizing Affymetrix Human Exon 1.0 ST v2 Arrays. Preprocessing included background adjustment, quantile normalization and summarization to the probeset level using the oligo package in R. Batch corrections were made using surrogate variable analysis. Gene annotations were sourced from GENCODE, with data summarized to gene-, transcript- and exon-level (see Supplemental Methods for more details). This led to expression values of 17 447 genes, 100 750 transcripts and 242 443 exons.

For Dataset 2a, single-nucleus RNA-seq libraries were generated from post-mortem human brain tissue using the 10X Genomics Chromium platform. Nuclei were isolated from brain tissue and processed according to standard protocols. Data were processed using established bioinformatics pipelines, including alignment using Cell Ranger. Subsequently, count matrices were processed using Scanpy, including filtering of cells based on counts and mitochondrial genes, normalization, log-transformation and clustering using highly variable genes. Cell type annotation was performed based on the expression of known marker genes for major neuronal and non-neuronal cell types (see Supplemental Methods for more details).

### Genotype data, imputation and polygenic risk scores (PRSs)

For Dataset 1, genotyping was conducted using Illumina Infinium Global Screening Arrays on genomic DNA extracted from postmortem cerebellar tissue. Quality control measures were applied using PLINK [27]. The study population primarily consists of individuals of European ancestry. Imputation of missing genotypes was performed with IMPUTE2 [28], utilizing the 1 000 Genomes Phase III reference panel, which includes individuals of European ancestry, as a reference. SNP coordinates are aligned with the hg19 genome assembly (totaling 9 164 462 SNPs; further details are available in the Supplemental Methods). Additionally, PRSs were calculated using PRSice-2 [29] with a significance threshold of 0.01 (additional information provided in the Supplemental Methods).

### Phenotypic data

Phenotypic data from Dataset 1, including age, sex, pH, postmortem interval (PMI), RNA integrity number (RIN), suicide status and cause of death (CoD; natural, violent or non-violent) were collected. Ancestry dimensions were calculated using multidimensional scaling based on genotypes.

### Differential expression analysis

For the differential expression analyses, all samples diagnosed with a psychiatric disorder were grouped as affected (n = 107). We used the *limma* version 3.42.2 package in R 3.6.1 for analysis. The effect of being diagnosed or not on gene expression differences was assessed, controlling for age, sex, pH, PMI, RIN, RIN^2^, suicide and CoD. Additionally, adjustments were made for the first four dimensions of genotype-defined ancestry (Dim1-Dim4) and one identified surrogate variable (SV1) not correlated with other model covariates. Differential expression analysis was conducted separately for each genetic level and only results with an FDR below 0.1 were considered for further analysis. For transcript- and exon-level expressions, only one exon or transcript per gene model was subjected to multiple testing correction, so as not to confound the correction of genes with hundreds of exons (Figure S1). The FDR was recalculated by: (1) generating p-values using *limma*, correcting for all covariates; (2) selecting the transcript or exon with the lowest p-value for each Ensembl gene ID to create a new expression matrix; and (3) recalculating the FDR using *limma* on this refined matrix. Differential expression analyses extended beyond diagnosis to include all listed covariates, adjusting for all other model covariates (as shown in Figure S3 and Figure S4A).

### Expression quantitative trait locus (eQTL) analysis

For the eQTL analysis, we utilized all imputed SNPs with a minor allele frequency (MAF) greater than 5% for robust statistical power, totaling 6 830 577 SNPs. The eQTLs were calculated using the additive linear model provided by MatrixEQTL [30], with adjustments for age, sex, pH, PMI, RIN, RIN^2^, suicide, CoD, Dim1-4 and SV1. The threshold for *cis*-eQTL significance was set at 0.05, with the physical distance criterion set to 1 Mb.

### Exon expression quantitative trait score (eQT-Score) analysis

To compute exon eQT-Scores, we followed the procedure similar to the one used for the differential expression analysis. In this approach, we replaced the diagnosis variable with PRS in a linear model, adjusting for age, sex, pH, PMI, RIN, RIN^2^, suicide, CoD, Dim1-4 and SV1. PRSs for psychiatric disorders (BD [31], cross disorder [32], MDD [33] and schizophrenia [3]) were derived from respective GWAS studies, while PRS for the non-psychiatric GWAS type 2 diabetes (T2D) [34] served as negative control. Calculations were conducted on the entire 169-subject sample. Additional methodological details are available in the Supplementary Methods section.

### Core gene set generation

To generate the core gene set (n = 110), we identified genes that were present in all three data categories: exon-level eQTL genes (n = 6 401) and joint exon eQT-Score genes (n = 11,102) from Dataset 1 and genes harboring rare variants (n = 231) from the SCHEMA consortium [8]. The overlap was performed using Ensembl gene IDs to ensure consistency across datasets.

### Enrichment analyses

We conducted enrichment analysis for eQTL SNPs (eSNPs) using public data from Ensembl Variant Effect Predictor (VEP) [35], the core 15-state model of chromatin in DLPFC from the Roadmap Epigenomics Project [36] and GWAS summary statistics for attention-deficit hyperactivity disorder (ADHD) [37], autism spectrum disorder (ASD) [38], bipolar disorder (BD) [31], cross-disorder meta-analysis (CDG) [32], educational attainment (EA) [39], major depressive disorder (MDD) [33], schizophrenia (SCZ) [3] and type 2 diabetes (T2D) [34]. Our background dataset comprised all eQTL SNPs overlapping with the GWAS datasets. To mitigate bias stemming from different minor allele frequency (MAF) distributions across GWAS and our cohort, we binned MAFs for each eSNP set (gene, transcript and exon-level eSNPs) and the background SNP set in increments of 0.05 from 0–1. We then conducted an enrichment analysis using 10,000 permutations, assessing overlap between randomly selected background SNPs and public datasets. An empirical p-value was generated based on the frequency of overlaps exceeding the actual overlap of the eSNP set with the public dataset. We calculated odds ratios (OR) by dividing the actual overlap by the mean of the resampled overlaps. ORs were considered significant if the associated empirical p-value was less than 0.05 (Tables S6i–v).

### KEGG pathway analysis

For the KEGG pathway analysis of exon-level differentially expressed genes and the core gene set, we utilized FUMA GENE2FUNC [40], excluding disease and drug treatment relations. Default parameters were applied, with exon-level based genes (n = 17 496) serving as the background list. Only significant pathways with an FDR ≤ 5% were considered for KEGG pathway enrichment.

### Cell type enrichment analysis

We performed cell type enrichment analysis using two snRNA-seq datasets (Datasets 2a–b) to identify cell types enriched among the genes with the highest mean expression of the core genes (n = 110). Core genes and all tested genes (n = 17 044) were mapped to single-nuclei expression data using Ensembl gene IDs [41]. Most core genes (106/102) and background genes (14 600/14 377 for Dataset 2a and Dataset 2b, respectively) were detected in the single-nuclei dataset. The cell type distribution of the top 25% cells based on mean expression value for core genes was compared to the distribution considering all background genes. Statistical significance of enrichment in each cell type was evaluated using Fisher’s exact test. Additionally, cell-type-specific analysis was conducted using WebCSEA [42] on snRNA-seq data from BA6 and BA10 (Dataset 2c) with 107 core genes detected, using a combined p-value threshold of 0.05.

## Results

### Exon-level gene expression in cortex is associated with psychiatric disorder diagnosis

Traditional gene expression analyses often overlook subtle molecular changes occurring at lower resolutions, such as differentially expressed exons, particularly in complex diseases like psychiatric disorders. To address this limitation, we conducted a comprehensive profiling of gene expression in postmortem DLPFC tissue from 169 individuals aged 18–87 years, using an exon array data analysis strategy (Figure S1). Our cohort comprised 15 individuals with BD, 24 with MDD, 68 with schizophrenia, and 62 controls with no psychiatric diagnosis (Table S1).

**Fig. 1:**
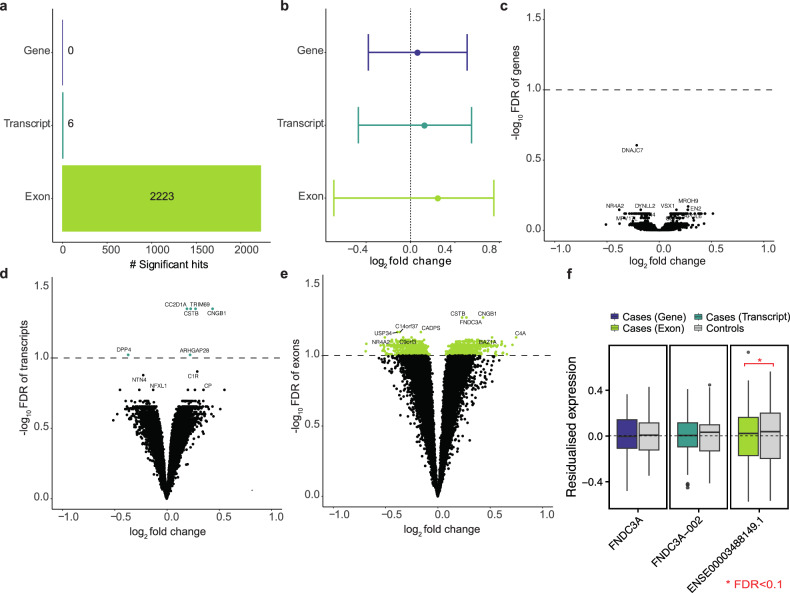
Differential expression analysis results. **a** Bar plot illustrating significantly differentially expressed genes at the gene-, transcript- and exon-level. **b** Forest plot displaying the log_2_ fold change (log_2_FC) range and median absolute log_2_FC (dot) of the 2 223 exon-level differentially expressed genes compared to transcript- and gene-level. Exon-level changes exhibit a larger magnitude (median absolute log_2_FC = 0.23, range of −0.69–0.75) than transcript- (median absolute log_2_FC = 0.11, range of −0.47–0.55) and gene-level (median absolute log_2_FC = 0.047, range of −0.38–0.51). Volcano plots depicting (**c**) gene-level, (**d**) transcript-level and (**e**) exon-level differential expression analysis outcomes. The x-axis represents log_2_FC, while the y-axis shows -log_10_(FDR). Significant hits (FDR < 0.1) are highlighted in turquoise for transcript-level and light green for exon-level differentially expressed gene hits. Among the exon-level differentially expressed genes, 51% (1 139 of 2 223 genes) and among the transcript-level differentially expressed genes, 83% (5 of 6 genes) are upregulated. **f** Boxplots illustrating the effect of diagnosis on *FNDC3A* expression, separately for each level: *FNDC3A* gene, *FNDC3A-002* transcript and *ENSE00003488* exon residualised expression for cases (purple, turquoise, or light green) and control subjects (gray). The x-axis indicates expression residuals and an asterisk indicates a significant multiple testing corrected p-value (FDR < 0.1).

We compared differential expression at gene-, transcript- and exon-levels. Grouping samples with BD, MDD and schizophrenia into a cross-disorder diagnosis, we observed no significant differential expression at the gene-level. However, at the transcript-level, six genes showed differential expression, whereas at the exon-level, 2 223 genes exhibited significant differential expression (Fig. 1a, Figure S4A and Table S2i–iii).

KEGG pathway analysis of exon-level differentially expressed genes revealed pathways related to cell-cell interactions, cell motility and organismal systems, with the extracellular matrix-receptor interaction (FDR = 7 × 10^−5^) and complement and coagulation cascade (FDR = 0.027) pathways primarily containing up-regulated genes (Figure S4B). In contrast, the axon guidance pathway (FDR = 0.046) showed predominantly down-regulated genes (Figure S4B).

Gene-level analysis did not yield any differentially expressed genes, confirming the importance of exon-level analysis. Six genes differentially expressed at the transcript-level were also differentially expressed at the exon-level. Further examination of the fold changes revealed a decrease in the magnitude of differential expression from exon- to transcript- to gene-level (Fig. 1b–e). We focused on fibronectin type III domain containing 3A (FNDC3A), an ECM-glycoprotein that plays vital roles during tissue repair, as a multi-exon gene, and found significant association with diagnosis in one specific exon (ENSE00003488149, FDR = 0.0539), while most exons showed no expression differences (Figs. 1f and S4E).

Our findings demonstrate that exon-level analysis captures molecular alterations in psychiatric cases, emphasizing the importance of sub-gene alterations typically overlooked by gene-level analysis.

### Common variants associated with changes in cortical gene expression are enriched for psychiatric cross-disorder GWAS traits

**Fig. 2:**
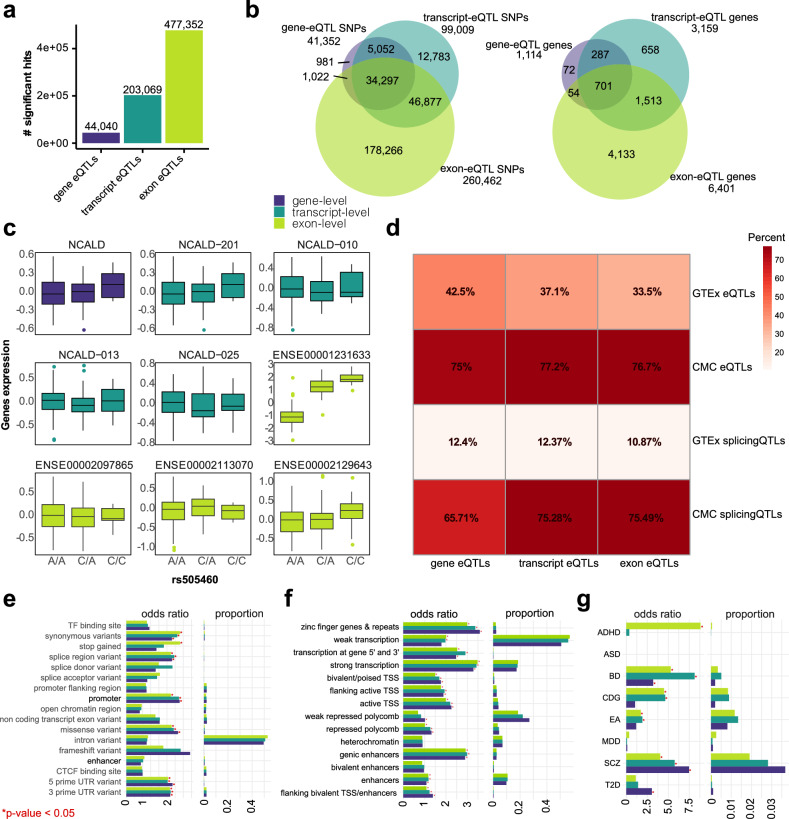
eQTL results and enrichment analysis of genomic features. **a** Bar plot showing significant eQTLs at the gene-, transcript- and exon-level. **b** Venn diagram illustrating the overlap of eQTL genes among all three levels. **c** Example association plot for rs505460-*NCALD* expression, displaying the effects of this variant on the expression of this gene at all three levels. rs505460 only has a visible influence on the expression of exon ENSE00001231633. **d** Overlap between genes from the three eQTL levels and eQTL and splicingQTL genes from the GTEx or CMC datasets in the human cortex. Genomic overlap between eSNPs of all three levels with (**e**) Ensembl Variant Effect Predictor (VEP) categories, (**f**) the 15-state model of the Roadmap Epigenomics Project measured in DLPFC and (**g**) various GWAS traits from the Psychiatric Genomics Consortium and non-psychiatric phenotypes as negative controls. The results are presented as bar plots showing odds ratios with significant p-values indicated by an asterisk (*p-value < 0.05), along with proportions of overlap with the original datasets. ADHD attention-deficit hyperactivity disorder, ASD autism spectrum disorder, BD bipolar disorder, CDG cross disorder meta-analysis, EA educational attainment, MDD major depressive disorder, SCZ schizophrenia, T2D type 2 diabetes.

We performed *cis*-eQTL analysis to identify genetic variants influencing gene expression independently of diagnosis and discovered 44 040 gene-eQTLs, 203 069 transcript-eQTLs and 477 352 exon-eQTLs at FDR < 5% (Fig. 2a, Figure S5A–C, Tables S3–S5). The number of uniquely identified SNPs and genes increased from gene- to exon-level analysis (Figs. 2b and S5D), with 65% of genes exclusively detected at the exon-eQTL level. The *calcium-binding gene neurocalcin delta* (*NCALD*) is an example of an exon-level-specific effect, where only one out of 13 exons (ENSE00001231633, FDR = 4 × 10^−45^) was differentially expressed in a variant-dependent manner (Fig. 2c). Notably, 49% of the exon-level differentially expressed genes (n = 1 090 out of 2 223 genes) were in common with exon-eQTL genes.

We validated our eQTLs by comparing them to prior PFC (BA9) eQTLs from GTEx v8 (425 donors) and the CMC (537 donors, including individuals with schizophrenia [11]) cohorts. The overlap with the GTEx data was lower compared to the disease-matched CMC cohort, with a high overlap (>75%) observed between our transcript- and exon-level eQTLs and CMC e/splicingQTLs, demonstrating the reliability of our eQTL identification (Fig. 2d).

We characterized our eQTLs at gene, transcript, and exon levels, finding eQTL (e)SNPs primarily located within intronic regions and enriched in various regulatory elements (Fig. 2e). Exon-level eSNPs showed specific enrichment for stop gains. Chromatin state analysis revealed associations with both active and repressive chromatin marks (Fig. 2f). Additionally, we observed significant enrichment of eSNPs at all three levels in GWAS risk variants for psychiatric disorders, including BD and schizophrenia (Fig. 2g), with exon-level eSNPs showing the largest overlap (Table S6iii). For detailed results, refer to Supplementary Results.

### Joint-SNP effects associated with cross-diagnostic disease risk influence cortical exon expression

**Fig. 3:**
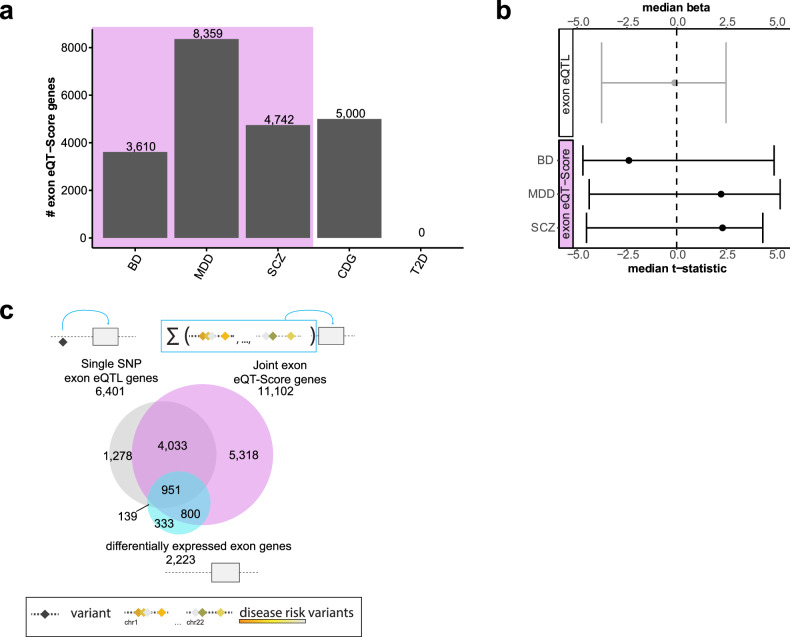
Joint-SNP effects on cortical expression. **a** Bar plot displaying the number of significant exon-level expression-polygenic risk score associations, also known as the exon expression quantitative trait score (exon eQT-Score). The y-axis represents the counts of exon eQT-Score genes, and the x-axis indicates the corresponding GWAS used in the eQT-Score calculation. **b** Forest plot illustrating the effect size of exon-level eQTL genes and exon eQT-Score genes. The y-axis shows the median beta or t-statistic and the x-axis displays the GWAS used in the eQT-Score calculation. Exon eQT-Score genes exhibited a larger effect size (median absolute schizophrenia t-stats = 2.55, range from −4.53–4.32, median absolute MDD t-stats = 2.46, range from −4.39–5.2, median absolute BD t-stats = 2.63, range from −4.81–4.89) compared to single-exon-level eQTL genes (median absolute beta = −0.1, range from −3.77–2.48, p-value Wilcoxon test < 2.2 × 10^−16^). **c** Venn diagram showing the overlap between exon eQTL genes (gray), the joint BD, MDD and SCZ GWAS exon eQT-Score genes (purple) and differentially expressed exon-level genes (blue). BD bipolar disorder, MDD major depressive disorder, SCZ schizophrenia, CDG cross disorder, T2D type 2 diabetes.

We next investigated the relationships between exons in BA9 and genomic loci identified in large psychiatric GWASs. To encode genetic disease risk, we employed PRS analysis to calculate a cumulative sum of risk variants per individual for BD, MDD, cross-disorder and schizophrenia GWASs. We focused on exon-level expression due to the robust signal in our cohort. We computed the exon expression quantitative trait (eQT)-Score by combining individual PRSs with exon-level expression data (Fig. 3a), following the same analysis approach used for differential gene expression (see Methods). The eQT-Score analyses revealed numerous genes associated with BD, MDD and schizophrenia (3 610-8 359) per eQT-Score, corresponding to a total of 11 102 unique genes and 16 199 exons (Table S8), hereafter referred to as the joint exon eQT-Score set, surpassing the number of exon-level genes associated with the diagnosis (2 223, c.f. Fig. 1b). This suggests that the comprehensive genetic disease architecture offers more informative insights than binary diagnosis codes. In contrast, the negative control GWAS for type 2 diabetes did not yield any hits.

The joint exon eQT-Score set, which included 78.8% of exon-level genes associated before with clinical diagnosis, indicated a substantial contribution to genetic disease risk. By comparing the joint-SNP approach to single-SNP eQTL results, we observed that 77.9% of single-exon-level eSNPs overlapped with the joint exon eQT-Score set. The joint-SNP approach exhibited larger effect sizes than the single-SNP approach (Fig. 3b). Additionally, the joint exon eQT-Scores showed differential expression for diagnosis (15.8%, n = 1 751 differentially expressed genes, Table S2iii) and exhibited single-SNP effects in cis (44.9%, n = 4 985 exon-level eQTL genes, Table S5), see Fig. 3c.

### Rare and common variants share risk for cross-diagnostic psychiatric disorders

**Fig. 4:**
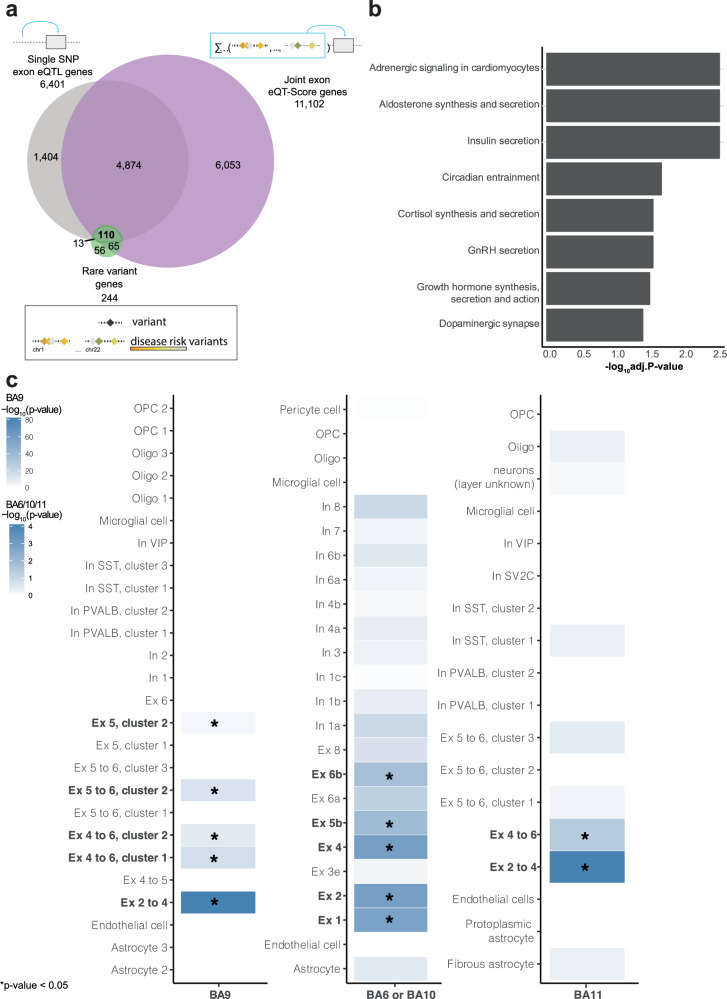
Genes disrupted by rare and common variants. **a** Venn diagram showing the overlap between single SNP exon eQTL genes (gray), joint exon eQT-Score genes (purple) and genes altered by rare variants in the SCHEMA consortium data [8] (green). **b** Bar plot illustrating the KEGG pathway enrichment analysis results for the core set of genes (n = 110). The top 10 pathways are visualized, with dark gray bars denoting statistically significant pathways. The significance threshold, set at FDR < 0.05 / -log_10_(adj.P-value)>1.3, is indicated by the dashed red line. The y-axis represents the enriched KEGG pathways, while the x-axis displays the -log10-transformed adjusted p-values (FDRs). **c** Heatmap depicting cell-type specificity for the core gene set of enrichment signals defined at the gene-level from the snRNA-seq data (BA11: Dataset 2a, BA9: Dataset 2b, BA6/10: Dataset 2c). The star indicates a significant enrichment p-value < 0.05. Ex excitatory neurons, In inhibitory neurons (identified based on the expression pattern of peptide genes: VIP, SST, SV2C and calcium-binding protein: gene PVALB), OPC oligodendrocyte precursor cell, Oligo oligodendrocyte.

Given that common variants explain only a small proportion of schizophrenia heritability, we incorporated rare variant data from the SCHEMA Consortium (exomes from whole blood of 24 248 schizophrenia cases and 97 322 controls without a psychiatric diagnosis) [8], identifying 244 schizophrenia-associated genes (231 in Dataset 1). We hypothesized that genes implicated by both common and rare variants represent core disease mechanisms. Common variants may have subtle effects, while rare variants can be disruptive; convergent findings highlight robust targets. Integrating common exon-level eSNP and joint-SNP effects with rare variant data, we identified 110 core genes (Fig. 4a, Table S9), prioritizing genes with multiple lines of genetic and transcriptomic evidence.

KEGG pathway analysis of these core set genes revealed significant enrichment of brain-related pathways, including Circadian entrainment (FDR = 0.020), Cortisol synthesis and secretion (FDR = 0.027), and Dopaminergic synapse (FDR = 0.038). Also, several other pathways showed enrichment, including hormone-related pathways (FDRs < 0.0298) and Adrenergic signaling in cardiomyocytes (FDR = 0.0028), as shown in Fig. 4b and Table S10. Examination of hormone production pathways (Aldosterone synthesis and secretion, Insulin secretion, Cortisol synthesis and secretion, GnRH secretion, and Growth hormone synthesis, secretion and action) revealed that each involved 4–6 genes from our dataset out of 63–117 pathway genes. Specifically, *CACNA1C*, a voltage-gated calcium channel involved in neuronal signaling and shown to increase risk of psychiatric disorders [43], was shared across multiple hormone-related pathways, suggesting a broader role of calcium signaling in neuronal and endocrine regulation. Pathway-specific genes such as *PCLO* from Insulin secretion and *DAGLA* from Aldosterone synthesis and secretion highlight distinct roles within their hormonal systems. These findings indicate a similar functional impact of rare and common variants in psychiatric disorders.

We finally studied the cell type specificity of the core genes by analyzing snRNA-seq data from BA9 (Dataset 2b) and comparing it to data from other cortical areas (BA6/10/11, Dataset 2a, c; see Supplementary Results). The core genes showed significant enrichment in excitatory neurons (layers 2–6) within BA9 and BA11 and replicated in snRNA-seq data from BA6 and BA10 cortices (Fig. 4c).

**Fig. 5:**
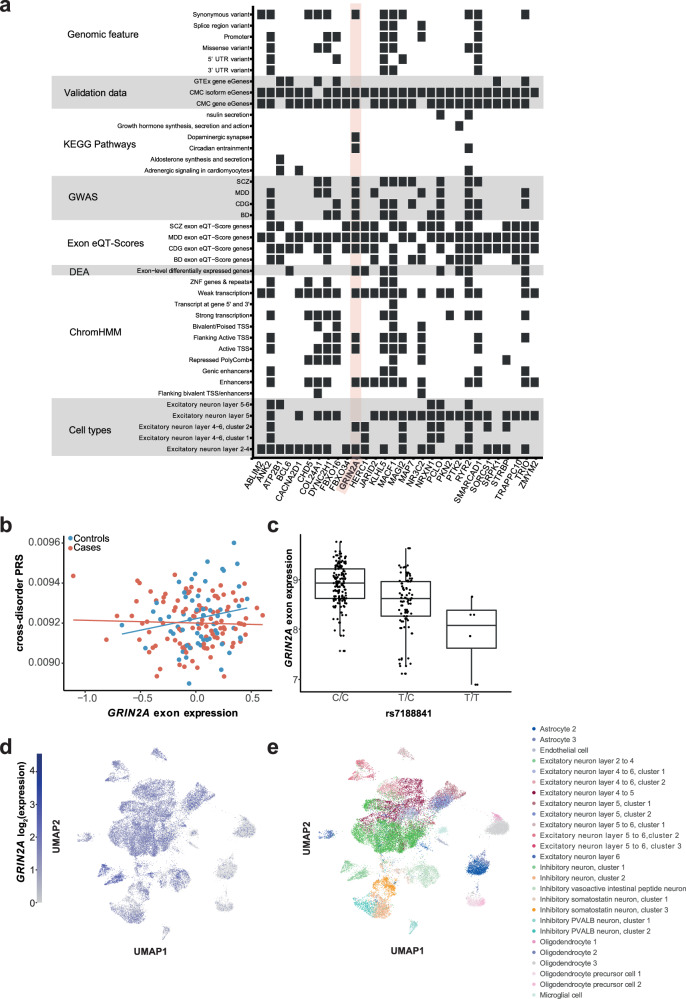
The missense gene *GRIN2A*, a core gene enriched in excitatory neurons. **a** Heatmap displaying 30 genes from the core gene set significantly enriched in at least one of the overrepresented BA9 cell types. The plot shows the datasets in which the genes are significant. **b** Association plot of cross-disorder PRS and ENSE00001323909.1 exon-level expression of *GRIN2A*, separated into cases and controls with mean residualized expression in control = 0.04 and cases = −0.02 (cross-disorder exon eQT-Score). The x-axis indicates expression residuals and the y-axis shows cross-disorder PRS values. **c** Box plot indicating the exon-level eQTL expression of *GRIN2A* exon ENSE00001323909.1 for rs1545099. The y-axis indicates expression residuals and the x-axis shows genotypes. **d** UMAP of the Glutamate Ionotropic Receptor NMDA Type Subunit 2A (*GRIN2A)* expression in BA9, where gray denotes minimal expression and blue represents high expression. **e** UMAP of the 26 clusters identified in BA9.

To gain deeper mechanistic insight, we conducted a cell type enrichment analysis (see Materials and Methods), which identified BA9 excitatory neuron layers 2–4, 5, 4–6 or 5–6 with p < 1 × 10^−50^ as the most significantly enriched cell types. Based on this analysis, we derived a subset of 30 genes from the core gene set specifically associated with these enriched excitatory neurons and examined their annotations and associations (Fig. 5a). The majority (87–97%) of these genes overlapped with previously identified cortical eQTL and splicingQTL genes from the CMC dataset. Furthermore, 50–67% of the exon-level eSNPs were enriched in weakly transcribed regions or enhancers. In addition, 90% of the genes were significantly associated with PRS for MDD, 73% for cross-disorder and 60% for schizophrenia. Interestingly, only nine (30%) of the genes overlapped with the differentially expressed exon-level genes. Among the highly annotated genes were *Ankyrin-2* (*ANK2*), *Ryanodine Receptor 2* (*RYR2*) and *Glutamate Ionotropic Receptor NMDA Type Subunit 2A* (*GRIN2A*), see Fig. 5a*. GRIN2A*, a key glutamate system gene, is enriched in excitatory neurons and linked to several psychiatric disorders (schizophrenia, BD, MDD and cross-disorder), actively-transcribed states and DLPFC enhancers. This gene included synonymous variants and was confirmed to be a cortical e/splicingQTL gene. *GRIN2A’s* top eQT-Score exon was down-regulated in patients, and controls had slightly greater mean cross-disorder PRS scores (mean = 0.04) compared to patients (mean = −0.02), see Fig. 1b. The presence of specific exons was associated with cross-disorder, MDD and schizophrenia PRSs. Furthermore, individuals with the T allele of SNP rs1545099 showed significant up-regulation of *GRIN2A* exon-level expression (Fig. 5c), which was mainly localized in excitatory neuron layers 2–4 and 4–6 (Fig. 5d, e).

## Discussion

Over 95% of multi-exon genes undergo alternative splicing, particularly in the brain [44], indicating its importance in neuronal development and function [45]. Splicing defects have been implicated in the pathogenesis of psychiatric disorders, including schizophrenia [46]. This study provides a comprehensive investigation of targeted exon-level gene expression in the human brain, emphasizing the importance of exon-level gene expression in the pathophysiology of psychiatric disorders. Unlike conventional methods that average expression across entire transcripts or genes, we precisely measured exons, leading to the identification of 2 223 genes with differentially expressed exons between psychiatric cases and controls (Fig. 1), showing larger fold changes than those observed at the gene- or transcript-level. This finding is consistent with previous research that has shown larger fold changes at the exon level compared to the gene level [21]. Moreover, we observed no significant main effects of diagnosis at the gene-level and only a few at the transcript-level (n = 6 genes). Our focus was on individuals with psychiatric disorders, specifically those with symptoms associated with the schizophrenia spectrum. This approach better mirrors the clinical and scientific reality of psychiatric disorders, capturing their complexity, dimensionality, and comorbidity [47]. Many of our identified differentially expressed genes at the transcript- and exon-level, such as *Cyclic Nucleotide Gated Channel Subunit Beta* 1 (*CNGB1*), *Cystatin B* (*CSTB)* and *Dipeptidyl peptidase 4* (*DPP4)*, have been previously linked to psychiatric disorders in various studies, including exome sequencing and GWAS of schizophrenia [3, 48–50].

By integrating transcriptome and genetic variation data, we found a significantly greater number of exon-level eQTLs, two times greater than at the transcript-level and ten times greater than at the gene-level (Fig. 2a). This finding is consistent with previous reports demonstrating the transcript-specificity of many eQTL signals, particularly those identified in brain tissue [21]. An example of an exon-level-specific eQTL is *Neurocalcin Delta* (*NCALD*), a brain-enriched protein associated with various neurological disorders (Fig. 2c). In genetic rat models of schizophrenia, *NCALD* expression was downregulated and in *NCALD* knockout mice, it was linked to adult hippocampal neurogenesis [51, 52]. Additionally, *NCALD* SNPs have been associated with bipolar disorder [53]. Interestingly, 75–77% of our significant exon-level eQTL genes in the present study overlapped with published postmortem eQTL genes, while 65–75% overlapped with splicingQTL genes, confirming the robustness of our findings and the importance of studying exon-specific expression. Variant effects from exon-level eQTLs highlight an accumulation of gene flanking regions and synonymous and missense variants. Moreover, exon-level eSNPs were enriched in stop-gained variants, indicating a potentially higher protein-damaging impact compared to transcript- or gene-level analysis. Despite the high polygenicity of psychiatric diseases and the distinction in variant sets between GWAS and eQTL studies [54], we found significant enrichment of our exon-level eSNPs in GWAS SNPs associated with ADHD, BD, cross-disorder, educational attainment and schizophrenia, providing further evidence for the shared genetic basis of psychiatric disorders. Although eQTL analysis provides valuable insights, it may not directly elucidate the underlying disease mechanisms involved. Instead, this finding underscores the significance of conducting gene expression analysis at the exon-level.

In our study, we employed a novel approach that combines polygenic risk scores (PRSs) with exon-level expression data (eQT-Scores) to gain deeper insights into the genetic foundations of psychiatric disorders. Psychiatric disorders are known for their intricate symptomatology and frequent co-occurrence, underscoring the need for a continuous, transdiagnostic perspective to better comprehend the complex genetic underpinnings of these conditions. With this approach we found that 79% of the genes associated with diagnosis share commonalities with genes implicated in genetic risk, while 81% of the genes related to genetic risk (eQT-Score genes) provide additional insights (Fig. 3c). This striking observation implies that genetic risk factors extend their influence on additional genetic loci beyond those directly associated with the disorders themselves. Furthermore, our eQT-Score approach encompasses information from multiple SNPs, offering a broader perspective, while traditional eQTL analysis often involves scrutinizing a single SNP at a time. This expanded scope resulted in the identification of 1.6–3.8 times more differentially expressed genes (Fig. 3a, c) and effect sizes that were 22.3–47.5 times greater than those observed at the single-exon-level in eQTL analysis (Fig. 3b). Our findings illustrate the importance of exon-level expression analysis and its integration with genetic risk through PRS, offering a deeper understanding of the genetic foundations of psychiatric disorders and the potential to enhance risk prediction, diagnosis, and therapeutic strategies. Further advances in larger sample sizes and incorporation of functional annotations such as cell type or epigenetic marks could enhance the results of exon eQT-Scores, leading to more accurate diagnostic tools in the future.

Additionally, we combined the effects of common and rare variants, acknowledging the limited impact of common variants and the potential significance of rare variants in psychiatric disorders. We analyzed rare coding variants from whole exomes from the SCHEMA consortium [8] and found mutations affecting the same genes as those identified for exon-level eQTLs (50% overlap, Fig. 4a), suggesting that shared loci are the basis for schizophrenia. Our findings, despite the complexities and shared genetic basis of psychiatric disorders, demonstrate robustness and consistency with larger studies, validating their reliability.

Integrating the various genetic models of the transdiagnostic psychiatric phenotype (eQTL, eQT-Score and rare variants) identified a core set of 110 genes representing a combination of rare and common risk factors for psychopathology. These genes are significantly enriched in several pathways, including those related to neurotransmission (dopaminergic synapse and adrenergic signaling in cardiomyocytes), circadian entrainment, as well as in the production of hormones (cortisol, insulin, aldosterone and GnRH) (Fig. 4b). Interestingly, adrenergic signaling, which plays a crucial role in the sympathetic nervous system and influences various cognitive and emotional processes [55], was also significantly enriched. This is an unexpected finding, considering the primary function of the DLPFC in higher cognitive processes. While the DLPFC does not directly innervate the heart, the enrichment of the adrenergic signaling in cardiomyocytes pathway may reflect shared regulatory mechanisms or indirect connections, potentially influenced by the higher prevalence of cardiometabolic conditions, such as diabetes and cardiovascular disease, in individuals with psychiatric disorders [56]. While increased striatal dopamine is associated with psychosis, evidence suggests prefrontal cortical dopamine deficits in schizophrenia. Animal studies support this, showing that prefrontal cortex dopamine lesions increase striatal dopamine, while dopamine agonists reduce it. Antipsychotics targeting D2 receptors further support dopamine’s involvement [57]. However, dopamine’s role in schizophrenia is complex and involves intricate interactions within and between brain regions. Circadian dysfunction is frequently observed in major psychiatric disorders [58] and is intricately connected to dopamine regulation, as dopamine entrains the master clock in the suprachiasmatic nuclei [59]. The suprachiasmatic nuclei also play a role in hormone secretion [60], aligning with our identification of genes related to cortisol, aldosterone, insulin and GnRH regulation, all exhibiting circadian rhythms [61, 62]. Notably, gene expression patterns in the prefrontal cortex exhibit diurnal rhythms [63], further supporting the relevance of circadian processes. These pathway findings highlight the potential involvement of multiple biological systems in the pathophysiology of psychiatric disorders.

Our core gene set was enriched in excitatory neuron subtypes of BA6, 9, 10 and 11. Notably, alterations in specific cortical layers of the DLPFC, particularly layers 2 and 3, have been associated with cognitive deficits in schizophrenia, including impairments in working memory and executive functions [64–66]. Cortical neurons seem vulnerable to the effects of stress in psychopathology [67], aligning with the implications of cortisol in our core gene set and its connection to circadian entrainment. Future studies focusing on single-nucleus omics will continue to unveil crucial cell types involved in psychiatric diseases [13].

To leverage multi-level information from common and rare genetics, as well as tissue transcriptomics (bulk exon array expression and snRNA-seq), we zoomed-in on a subset of core genes (n = 30 genes, Fig. 5a). We based this selection on comprehensive evidence from functional annotations and single-cell-type enrichment. Notably, *Glutamate Ionotropic Receptor NMDA Type Subunit 2A* (*GRIN2A*) has emerged as a standout gene that encodes the NMDA receptor NR2B subunit, regulating neuronal excitability. It has been strongly associated with schizophrenia in a recent large exome-sequencing study [8]. NMDA receptor hypofunction and glutamate dysregulation are consistently implicated in postmortem brain studies examining the cortex in schizophrenia patients and are closely linked to dopamine neurotransmission and dysfunction [66]. The NMDA receptor composition switches during development from NR2B to NR2A (*GRIN2A*) subunit dominance, influencing cortical circuit maturation and NMDA receptor hypofunction [68]. In-depth analysis of the core gene set may provide novel insights into schizophrenia and reveal common foundations across psychiatric disorders.

While postmortem studies provide only a snapshot of the brain at the time of death, they enable examination of brain pathology at cellular and molecular resolution, which is not possible with current imaging modalities in living people [69]. To account for possible confounding factors associated with the study of postmortem human brain tissues, we included demographic and clinical variables such as PMI, RIN and pH to ensure that the results obtained were related to pathology and not tissue quality characteristics. Additionally, while microarrays are informative, they may be less sensitive than RNA-seq methods. Future RNA-seq studies at the single-cell level may offer deeper insights.

It is worth noting that the cohort composition of our primary cohort (Dataset 1, comprising mainly schizophrenia cases (n = 68) with fewer cases of major depressive disorder (n = 24) and bipolar disorder (n = 15)) is biased towards schizophrenia diagnoses. This imbalance should be kept in mind when interpreting our findings, as some effect may be disproportionately influenced by this diagnostic group. Furthermore, Dataset 2, comprising a relatively small sample size, may limit the statistical power and generalizability of the findings from this dataset. These considerations highlight areas where future research can be improved based on the insights gained from this study. In addition, to ensure a sufficient number of significantly differentially expressed genes were identified, a less stringent threshold of FDR < 0.1 was employed. This threshold, while less stringent than the conventional FDR < 0.05, still maintains a relatively stringent level of statistical significance.

Overall, our study highlights the importance of examining exon-level gene expression directly in the human brain and integrating multi-level datasets to uncover crucial combined genetic risk and diagnosis-related gene sets with pathological relevance. This approach pointed to cortical excitatory neurons as a potentially crucial cell type in psychopathology and identified key genes such as *GRIN2A* as potential targets for further research and treatment development in psychiatric disorders.

## Supplementary information

Supplementary information

Supplementary Table 1

Supplementary Table 2i

Supplementary Table 2ii

Supplementary Table 2iii

Supplementary Table 3

Supplementary Table 4

Supplementary Table 5

Supplementary Table 6i

Supplementary Table 6ii

Supplementary Table 6iii

Supplementary Table 6iv

Supplementary Table 6v

Supplementary Table 7i

Supplementary Table 7ii

Supplementary Table 8

Supplementary Table 9

Supplementary Table 10

## Data Availability

The expression data of Dataset 1 can be accessed on the Gene Expression Omnibus (GEO) under the accession number GSE208338. However, access to the genotypes of Dataset 1 requires an approved request. The snRNA-seq data of Dataset 2 are also available on GEO. Specifically, the Dataset 2a can be found under the accession number GSE205642, the Dataset 2b under GSE144136 and the Dataset 2c under GSE97930. These GEO accessions provide access to the respective snRNA-seq data sets for further exploration and analysis. Code for the analysis is available at GitHub: https://github.com/cellmapslab/PostmortemBrainAnalysis. The web links for the publicly available datasets used in the study are as follows: SCHEMA results to integrate rare coding variants: https://schema.broadinstitute.org/downloads, 15-core state model of chromatin from Roadmap Epigenomics Roadmap (ChromHMM v1.10) for functional annotation of eSNPs: https://egg2.wustl.edu/roadmap/web_portal/chr_state_learning.html#core_15state, CommonMind Consortium for result comparison: http://CommonMind.org, Current NetAffx probeset annotation file for the Affymetrix HuEx 1.0 ST v2 microarray: http://www.affymetrix.com/Auth/analysis/downloads/na36/wtexon/HuEx-1_0-st-v2.na36.hg19.probeset.csv.zip, DIAbetes Genetics Replication And Meta-analysis (DIAGRAM) Consortium for the type 2 diabetes (T2D) GWAS summary statistic: https://diagram-consortium.org/downloads.html, GENCODE release 19 (GRCh37.p13) annotation file: http://ftp.ebi.ac.uk/pub/databases/gencode/Gencode_human/release_19/gencode.v19.annotation.gtf.gz, Genotype-Tissue Expression (GTEx) V8 for annotation/enrichment analysis and result comparison: https://www.gtexportal.org/home/datasets, Human Ageing Genomic Resources (HAGR) for known human aging genes: https://genomics.senescence.info/genes/human_genes.zip, National Center for Biotechnology Information (NCBI) for GRCh37/hg19 reference genome of the index-patient rare variant dataset: http://www.ncbi.nlm.nih.gov/assembly/2758/, Psychiatric Genomics Consortium (PGC) for GWAS summary statistics of psychiatric disorders: https://www.med.unc.edu/pgc/, Social Science Genetic Association Consortium (SSGAC) for the educational attainment (EA) GWAS summary statistic: https://www.thessgac.org/data, Web-based Cell-type-Specific Enrichment Analysis (WebCSEA): https://bioinfo.uth.edu/webcsea/.
