# Supplementary material for: Exon-variant interplay and multi-modal evidence identify endocrine dysregulation in severe psychiatric disorders impacting excitatory neurons
Source: Transl Psychiatry. 2025 Apr 19;15:153. doi: 10.1038/s41398-025-03366-8 (PMC12009313; doi:10.1038/s41398-025-03366-8)
Supplement: Supplementary file 1 — Supplementary information [file 41398_2025_3366_MOESM1_ESM.docx]

# Supplementary appendix - contents

[**Supplementary appendix - contents 1**](#_wgq2qko3h8b8)

[**Supplementary Results 1**](#_3se2jxwqbps3)

[1. Covariate analysis: exploring differential exon-level expression beyond diagnosis 1](#_auomd2ngd6hk)

[2. Genomic locations, regulatory features, and psychiatric cross-disorder GWAS enrichment of cortical eQTLs 1](#_w6rk1crwly3z)

[3. Cell type specificity of core genes in BA9 and comparison with other cortical areas 2](#_rbccn6slog0p)

[**Supplementary Materials and Methods 2**](#_xpzub36ody6c)

[1. Gene expression data 2](#_4f1mdlm)

Dataset 1: Exon arrays in BA9 2

[Dataset 2: snRNA-seq 3](#_798u4uk31ud0)

[Nuclei extraction and library preparation (Dataset 2a, BA11): 3](#_nuo9f2p0731y)

[Sequence Alignment, Filtering, Normalisation, Clustering and Cell type assignment (Dataset 2a, 3](#_44sinio)

[BA11): 3](#_19g9e41rn0fw)

[Filtering, Normalisation, Clustering (Dataset 2b, BA9): 4](#_z337ya)

2. Genotype data, imputation and PRS in Dataset 1 4

3. Phenotype data in Dataset 1 4

[**Supplementary References 5**](#_m43souv8kslq)

[**Supplementary Figures and Legends 7**](#_3tbugp1)

[**Supplementary Table Legends 12**](#_nmf14n)

# Supplementary Results

## Covariate analysis: exploring differential exon-level expression beyond diagnosis

In addition to diagnosis, we explored the differences in other covariates to understand the potential information gain at the sub-gene resolution (Figure S2,3 and S4A). Genetic ancestry, specifically captured by one ancestry dimension (Dim2) of the genotype data, exhibited notable variations at the gene-level. Moreover, age and pH exerted strong influences on gene expression. These findings collectively suggest that the observed exon-level differences are primarily associated with the diagnosis rather than reflecting a global effect on brain expression.

## Genomic locations, regulatory features, and psychiatric cross-disorder GWAS enrichment of cortical eQTLs

We characterized the genomic locations and regulatory features of our eQTLs to gain insights into their properties. Across all three levels, the majority of eQTL (e)SNPs were located within intronic regions and exhibited significant enrichment for various regulatory elements, including synonymous variants, splice regions, promoters, missense, 5' and 3' UTR variants ([Figure 2](https://docs.google.com/document/d/19QAxj0k0rbFBhr6zlDHNVlTxRvM33_dMhAjsPmma3dc/edit#bookmark=id.4k668n3)E). Notably, exon-level eSNPs showed specific enrichment for stop gains. Integrative analysis of DLPFC chromatin states using ChromHMM revealed that eSNPs at all three levels were associated with both active and repressive chromatin marks. Gene-level eSNPs exhibited a particular enrichment for weak repressed polycomb elements ([Figure 2](https://docs.google.com/document/d/19QAxj0k0rbFBhr6zlDHNVlTxRvM33_dMhAjsPmma3dc/edit#bookmark=id.4k668n3)F).

Next, we examined the association of identified eSNPs with genetic risk for psychiatric disorders by testing their enrichment among nominal significant variants from large-scale GWAS studies ([Figure 2](https://docs.google.com/document/d/19QAxj0k0rbFBhr6zlDHNVlTxRvM33_dMhAjsPmma3dc/edit#bookmark=id.4k668n3)G). Significant enrichment of eSNPs was observed at all three levels, specifically associated with schizophrenia and BD. Transcript- and exon-level eSNPs showed significant enrichment in cross-disorder and educational attainment GWAS, while only exon-level eSNPs were enriched for attention deficit hyperactivity disorder (ADHD). These findings suggest that the gene-, transcript-, and exon-level eQTL hits are influenced by the genetic architecture of psychiatric diseases, with exon-level data showing the largest overlap ([Table S](#sta_prs)6).

## Cell type specificity of core genes in BA9 and comparison with other cortical areas

To characterize the cell type specificity of these core genes, we used snRNA-seq data from the human BA9 (DLPFC, Dataset 2b) and compared this to other cortical areas (BA6/10/11). Among the 26 delineated cell type clusters from BA9 (Dataset 2b, n=17) and the 18 cell type clusters from BA11 (orbitofrontal cortex, Dataset 2b, n=2) the core genes were substantially enriched in excitatory neurons (layers 2-6, Figure 4C). We replicated the enrichment of our core genes in excitatory neurons with Web-based Cell-type-Specific Enrichment Analysis of Genes (WebCSEA) [^1^](https://paperpile.com/c/1nD4ZF/4dtvb) in snRNA-seq data from BA6 (premotor, n=6) and BA10 (frontal pole, n=6) cortices (Figure 4C).

# Supplementary Materials and Methods

## Gene expression data

### Dataset 1: Exon arrays in BA9

RNA preparation and expression array processing were carried out as described previously [^2^](https://paperpile.com/c/1nD4ZF/C9Wku). Briefly, total RNA was isolated from ∼100 mg frozen gray matter using 1.0 ml TRIzol reagent (Life Technologies, Scoresby, VIC, Australia). After homogenization and phase separation, the aqueous phase was added to an equal volume of 70% ethanol. RNA isolation was performed with RNeasy minikits (Qiagen, Cat. No. 74104, Chadstone Centre, VIC, Australia), with all samples treated with DNase using column digestion. DNA contamination was ruled out by PCR using specific primers for genomic DNA. RNA quantity and quality were analyzed by spectrophotometry (NanoDrop; Thermo Fisher Scientific Australia, Scoresby, VIC, Australia) and by determining RNA Integrity Numbers (RINs) using an Agilent 2100 bioanalyzer (Agilent Technologies, Santa Clara, CA, USA) and all samples with RIN ≥ 6.00 were used for further analyses with Affymetrix Human Exon 1.0 ST v2 Arrays according to the manufacturer’s instructions (Affymetrix, Santa Clara, CA, USA). Following hybridization, chips were scanned and the fluorescent signals converted into a DAT file for quality control. Finally, cell intensity (CEL) files were generated for further analyses.

Reading the raw data CEL files and storing the 6,553,600 microarray probes was performed using the *oligo* version 1.50.0 package [^3^](https://paperpile.com/c/1nD4ZF/FjNKz) in R 3.6.1. For background adjustment, quantile normalization and summarization (using median-polish) to probeset level *oligos* Robust Multichip Average (RMA) algorithm performing all three steps at once was used. Probesets without any start or stop information or labeled as controls in the current NetAffx annotation file for the Human Exon 1.0 ST v2 Array, downloaded from the Affymetrix support website, were removed from the dataset. Cross-hybridized probesets (*number_cross_hyb_probes* ≥ 1), composed of RNA target sequences binding to short DNA probes that are not exactly their complement, and probesets lying on the X, Y and M chromosome were also excluded from the dataset. To check the quality of the data, we analyzed the sample generation dates of the raw data as well as the log_2_-intensities, principal components and sample to sample distances for the raw data and the annotated data at gene-, transcript- and exon-level in R 3.6.1.

*SVA* version 3.34.0 [^4,5^](https://paperpile.com/c/1nD4ZF/8kjFa+WkL1Y) package in R 3.6.1 was used for batch correction of known and hidden batches. Batch correction was conducted at probeset level before annotation and summarization to different genetic levels. We first removed the five known batches with *SVA’s* *ComBat* function and then applied surrogate variable (SV) analysis to remove hidden effects. One SV was found, which was the only one explaining high variance in the expression dataset ([Figure S3](#sfi_covariates)). Gene annotations were downloaded from GENCODE [^6^](https://paperpile.com/c/1nD4ZF/xVZe7) release 19, which is the current version for the human hg19 (GRCh37) genome built on Ensembl version 74. Only protein-coding genes manually annotated by the Human and Vertebrate Analysis and Annotation (HAVANA) team were considered.

Summarization to gene, transcript, and exon-level was performed as follows: First, an annotation dataset was generated, where the GENCODE and the Affymetrix probeset information were merged by overlapping the locations in both datasets using the mergeByOverlaps function of the *GenomicAlignments* version 1.22.1 package in R 3.6.1. This annotation dataset was filtered for probesets, where at least one gene symbol correlated in both merged files and no multiple annotations were found. Second, for each sample the median of all expression values across all probes containing the same Ensembl ID was calculated. Multiple gene mappings were removed from all datasets. Some genes, transcripts and exons shared the exact same expression values in all samples leading to identical rows with different assigned IDs. This happens due to overlapping gene, transcript or exon locations. Instead of completely removing all identical rows, one row was kept and the different gene, transcript or exon IDs were merged into one ID. This led to expression values of 17,447 genes, 100,750 transcripts and 242,443 exons (all based on Ensembl IDs).

### Dataset 2: snRNA-seq

#### *Nuclei extraction and library preparation (Dataset 2a, BA11):*

Nuclei were isolated using an adapted version of a previously published protocol [^7^](https://paperpile.com/c/1nD4ZF/6afny). Briefly, frozen, dissected brain samples (50-60 mg) were dounce-homogenized in 1 ml nuclei extraction buffer (0.32 M Sucrose, 3 mM Mg(Ac)_2_, 5 mM CaCl_2_, 0.1 mM EDTA, 10 mM TrisHCl pH 8.1, 0.1% IGEPAL CA-630, 40 U/ml RiboLock RNase-Inhibitor (ThermoScientific)) on ice. Homogenate was layered onto 1.8 ml of sucrose cushion (1.8 M Sucrose, 3 mM Mg(Ac)_2_, 10 mM TrisHCl pH 8.1) and ultra-centrifuged at 28100 rpm for 2.5 hours at 4°C. Supernatant was removed and nuclei pellet was resuspended in 100 µl resuspension buffer (1X PBS, 3 mM Mg(Ac)_2_, 5 mM CaCl_2_, 1% BSA, 40 U/ml RiboLock RNase-Inhibitor). Nuclei suspension was filtered through a cell strainer cap. Nuclei were stained with DAPI 1:1000 and counted using a hemocytometer. Libraries for snRNA-seq were prepared following the user guide of 10X Genomics (Chromium Single Cell 3’ Reagents kit v3) with a target recovery of 10,000 nuclei per sample. Libraries were pooled equimolarily and were treated with Illumina Free Adapter Blocking Reagent before sequencing on the NovaSeq 6000 System (Illumina, San Diego, California, USA).

#### *Sequence Alignment, Filtering, Normalisation, Clustering and Cell type assignment (Dataset 2a,*

#### *BA11):*

Sequence reads were demultiplexed using the sample index, aligned to a pre-mRNA reference and UMI were counted after demultiplexing of nuclei barcodes using Cell Ranger v3.1.0. Count matrices were further processed using Scanpy v1.4.4 [^8^](https://paperpile.com/c/1nD4ZF/3DIfS). Count matrices of the two individuals were combined. Nuclei were filtered according to counts, minimum genes expressed and % of mitochondrial genes (Max counts > 50,000, Min counts < 1,000, Min genes > 400, Mito % ≥ 10). Genes expressed in < 20 nuclei were removed. Data were normalised and log-transformed using Scran [^9^](https://paperpile.com/c/1nD4ZF/dUenq). Embeddings were created using BBKNN [^10^](https://paperpile.com/c/1nD4ZF/B0HnG) and Louvain clustering [^11^](https://paperpile.com/c/1nD4ZF/pg4hR) using highly variable genes was applied for clustering. One cluster was excluded from the analysis due to highly variable genes being driven by or containing several MT-genes. Cell types were assigned to clusters based on marker gene expression as follows (Nagy et al. [^12^](https://paperpile.com/c/1nD4ZF/zd7u1) and Velmeshev et al. [^13^](https://paperpile.com/c/1nD4ZF/FXcKb)): Excitatory neurons: *SATB2, SLC17A7*, Layers: L2-4: *CUX2, THSD7A, L4-6: RORB, POU6F2, TSHZ2, RXFP1, L5-6: ETV1, KCNK2, PCP4*, Inhibitory neurons: *GAD1, GAD2*, Inhibitory neuron subtypes: In_PVALB: *PVALB*, In_SST: *SST*, In_VIP: *VIP, CALB2*, In_SV2C: *SV2C*, fibrous astrocytes (Astro_FB): high *GFAP, TNC, AQP4, GJA1*, protoplasmic astrocytes (Astro_PP): high *SLC1A2, AQP4, GJA1,* Microglia: *CD74, P2RY12, C3, CX3CR1,* Oligodendrocyte precursors (OPCs): *PCDH15, PDGFRA, OLIG1*, Oligodendrocytes (Oligo): *PLP1, MBP, MOBP, MOG,* Endothelial cells (Endo): *CLDN5, FN1, FLT1*.

#### *Filtering, Normalisation, Clustering* *(Dataset 2b, BA9):*

Nuclei extraction, library preparation and sequence alignment for this dataset are described in Nagy et al. [^12^](https://paperpile.com/c/1nD4ZF/zd7u1). Filtering of cells and cell type labels for downstream analyses were taken over from this processing. Raw counts of healthy donors were processed with Scanpy v1.4.4 [^8^](https://paperpile.com/c/1nD4ZF/3DIfS). Data were normalized and log-transformed, followed by Louvain clustering [^11^](https://paperpile.com/c/1nD4ZF/pg4hR) using highly variable genes.

## Genotype data, imputation and PRS in Dataset 1

Genotyping was conducted using Illumina Infinium Global Screening Arrays on 100 ng genomic DNA extracted from postmortem cerebellar tissue obtained from 169 subjects (107 cases and 62 controls, see [Table S1](#sta_demographics)) as previously described [^14,15^](https://paperpile.com/c/1nD4ZF/xQQld+NCykO). Quality control (QC) was conducted in PLINK 1.90b6.6 [^16^](https://paperpile.com/c/1nD4ZF/UV9hr). QC steps on samples included removal of individuals with a missing rate > 2%, cryptic relatives (PI-HAT > 0.0125), an autosomal heterozygosity deviation (|F_het_| > 4 SD) and genetic outliers (distance in the ancestry components from the mean > 4 SD). QC steps on variants included removal of variants with a call rate < 98%, a MAF < 1%, and HWE test p-values ≤ 10^-6^. Furthermore, variants on non-autosomal chromosomes were excluded. Imputation was performed with IMPUTE2, following phasing in SHAPEIT, using the 1,000 genomes phase III reference panel. QC of imputed probabilities was conducted in QCTOOL v1.5 64-bit. Imputed SNPs were excluded if MAF < 1%, HWE test p-values ≤ 10^-6^, or an INFO metric < 0.6. SNP coordinates are given according to hg19 (n=9,164,462 SNPs).

PRS for all 169 individuals were calculated using PRSice-2 v2.2.11.b (14th Oct 2019) [^17^](https://paperpile.com/c/1nD4ZF/62icf) using an empirical p-value threshold of 0.01. We chose this threshold because it represents the median of the best p-value thresholds given in the PRSice-2 summary files (min=0.0002, max=0.14, median=0.01, mean=0.04) and it also allows us to better compare the PRS calculated with the different GWAS. As input, we used the hard-called imputed and quality controlled genotypes of Dataset 1 as target, imputed genotypes from an independent cohort (recMDD [^18^](https://paperpile.com/c/1nD4ZF/tFW5j), n=1,774 Caucasian individuals) as LD reference and eight different publicly available GWAS summary statistics as base dataset. Polygenic risk was measured for different psychiatric disorders using the following GWAS summary statistics of the Psychiatric Genomics Consortium (PGC): bipolar disorder (BD) [^19^](https://paperpile.com/c/1nD4ZF/bG2O8), cross disorder (CDG) [^20^](https://paperpile.com/c/1nD4ZF/Br1vk), major depressive disorder (MDD) [^21^](https://paperpile.com/c/1nD4ZF/IHPQI) and schizophrenia (SCZ) [^22^](https://paperpile.com/c/1nD4ZF/z6H6N). We additionally generated PRS for a non-psychiatric GWAS as negative controls: the DIAbetes Genetics Replication And Meta-analysis (DIAGRAM) Consortium for type 2 diabetes (T2D) [^23^](https://paperpile.com/c/1nD4ZF/J0X3Y) ([Table S7](#sta_prs)i). P-value, pseudo-R^2^ based on Cox & Snell [^24^](https://paperpile.com/c/1nD4ZF/K385Q) and Cragg & Uhler (Nagelkerke) [^25^](https://paperpile.com/c/1nD4ZF/2N30J) approach were calculated using a fitted linear model approach and the Nagelkerke function of the *rcompanion* version 2.3.25 package in R ([Table S7](#sta_prs)ii).

## Phenotype data in Dataset 1

For three samples, no pH value was given, so we assigned them the mean value of all pH values from all other samples. We calculated the first ten ancestry dimensions (Dim1-10) using multidimensional scaling (MDS) based on raw Hamming distances in PLINK. Samples did not group into different subpopulations, although some samples were known to be of Asian (n=5) and mixed, Asian and Caucasian (n=1), origin, while the majority (n=123) were of Caucasian descent ([Figure S2](#sfi_covariates)). Only the Eigenvalue of the first four genetic ancestry dimensions (Dim1-4) changed verifiably, so only these were used in further analyses. Based on Stenbacka et al. [^26^](https://paperpile.com/c/1nD4ZF/pZfzo), the cause of death was divided into three categories: 1) "natural" for all natural causes, e.g. ischemic heart disease or pneumonia, 2) "violent" for violent suicides or accidents, such as hanging, drowning or a car accident, and 3) "non-violent" for all types of poisoning. A visual overview of all phenotypes is given in [Figure S](#sfi_covariates)3.

# Supplementary References

1 [Dai Y, Hu R, Liu A, Cho KS, Manuel AM, Li X *et al.* WebCSEA: web-based cell-type-specific enrichment analysis of genes. *Nucleic Acids Res* 2022. doi:](http://paperpile.com/b/1nD4ZF/4dtvb)[10.1093/nar/gkac392](http://dx.doi.org/10.1093/nar/gkac392)[.](http://paperpile.com/b/1nD4ZF/4dtvb)

2 [Scarr E, Udawela M, Thomas EA, Dean B. Changed gene expression in subjects with schizophrenia and low cortical muscarinic M1 receptors predicts disrupted upstream pathways interacting with that receptor. *Mol Psychiatry* 2018; **23**: 295–303.](http://paperpile.com/b/1nD4ZF/C9Wku)

3 [Carvalho BS, Irizarry RA. A framework for oligonucleotide microarray preprocessing. *Bioinformatics* 2010; **26**: 2363–2367.](http://paperpile.com/b/1nD4ZF/FjNKz)

4 [Johnson WE, Li C, Rabinovic A. Adjusting batch effects in microarray expression data using empirical Bayes methods. *Biostatistics* 2007; **8**: 118–127.](http://paperpile.com/b/1nD4ZF/8kjFa)

5 [Leek JT, Storey JD. Capturing heterogeneity in gene expression studies by surrogate variable analysis. *PLoS Genet* 2007; **3**: 1724–1735.](http://paperpile.com/b/1nD4ZF/WkL1Y)

6 [Frankish A, Diekhans M, Ferreira A-M, Johnson R, Jungreis I, Loveland J *et al.* GENCODE reference annotation for the human and mouse genomes. *Nucleic Acids Res* 2019; **47**: D766–D773.](http://paperpile.com/b/1nD4ZF/xVZe7)

7 [Matevossian A, Akbarian S. Neuronal nuclei isolation from human postmortem brain tissue. *J Vis Exp* 2008. doi:](http://paperpile.com/b/1nD4ZF/6afny)[10.3791/914](http://dx.doi.org/10.3791/914)[.](http://paperpile.com/b/1nD4ZF/6afny)

8 [Wolf FA, Angerer P, Theis FJ. SCANPY: large-scale single-cell gene expression data analysis. *Genome Biol* 2018; **19**: 15.](http://paperpile.com/b/1nD4ZF/3DIfS)

9 [Lun ATL, McCarthy DJ, Marioni JC. A step-by-step workflow for low-level analysis of single-cell RNA-seq data with Bioconductor. *F1000Res* 2016; **5**: 2122.](http://paperpile.com/b/1nD4ZF/dUenq)

10 [Polański K, Young MD, Miao Z, Meyer KB, Teichmann SA, Park J-E. BBKNN: fast batch alignment of single cell transcriptomes. *Bioinformatics* 2020; **36**: 964–965.](http://paperpile.com/b/1nD4ZF/B0HnG)

11 [Blondel VD, Guillaume J-L, Lambiotte R, Lefebvre E. Fast unfolding of communities in large networks. Journal of Statistical Mechanics: Theory and Experiment. 2008; **2008**: P10008.](http://paperpile.com/b/1nD4ZF/pg4hR)

12 [Nagy C, Maitra M, Tanti A, Suderman M, Théroux J-F, Davoli MA *et al.* Single-nucleus transcriptomics of the prefrontal cortex in major depressive disorder implicates oligodendrocyte precursor cells and excitatory neurons. *Nat Neurosci* 2020; **23**: 771–781.](http://paperpile.com/b/1nD4ZF/zd7u1)

13 [Velmeshev D, Schirmer L, Jung D, Haeussler M, Perez Y, Mayer S *et al.* Single-cell genomics identifies cell type–specific molecular changes in autism. Science. 2019; **364**: 685–689.](http://paperpile.com/b/1nD4ZF/FXcKb)

14 [Strauss WM. Preparation of Genomic DNA from Mammalian Tissue. Current Protocols in Neuroscience. 2001. doi:](http://paperpile.com/b/1nD4ZF/xQQld)[10.1002/0471142301.nsa01hs06](http://dx.doi.org/10.1002/0471142301.nsa01hs06)[.](http://paperpile.com/b/1nD4ZF/xQQld)

15 [Scarr E, Cowie TF, Kanellakis S, Sundram S, Pantelis C, Dean B. Decreased cortical muscarinic receptors define a subgroup of subjects with schizophrenia. *Mol Psychiatry* 2009; **14**: 1017–1023.](http://paperpile.com/b/1nD4ZF/NCykO)

16 [Chang CC, Chow CC, Tellier LC, Vattikuti S, Purcell SM, Lee JJ. Second-generation PLINK: rising to the challenge of larger and richer datasets. *Gigascience* 2015; **4**: 7.](http://paperpile.com/b/1nD4ZF/UV9hr)

17 [Choi SW, O’Reilly PF. PRSice-2: Polygenic Risk Score software for biobank-scale data. *Gigascience* 2019; **8**. doi:](http://paperpile.com/b/1nD4ZF/62icf)[10.1093/gigascience/giz082](http://dx.doi.org/10.1093/gigascience/giz082)[.](http://paperpile.com/b/1nD4ZF/62icf)

18 [Muglia P, Tozzi F, Galwey NW, Francks C, Upmanyu R, Kong XQ *et al.* Genome-wide association study of recurrent major depressive disorder in two European case-control cohorts. *Mol Psychiatry* 2010; **15**: 589–601.](http://paperpile.com/b/1nD4ZF/tFW5j)

19 [Mullins N, Forstner AJ, O’Connell KS, Coombes B, Coleman JRI, Qiao Z *et al.* Genome-wide association study of more than 40,000 bipolar disorder cases provides new insights into the underlying biology. *Nat Genet* 2021; **53**: 817–829.](http://paperpile.com/b/1nD4ZF/bG2O8)

20 [Cross-Disorder Group of the Psychiatric Genomics Consortium. Genomic Relationships, Novel Loci, and Pleiotropic Mechanisms across Eight Psychiatric Disorders. *Cell* 2019; **179**: 1469–1482.e11.](http://paperpile.com/b/1nD4ZF/Br1vk)

21 [Howard DM, Adams MJ, Clarke T-K, Hafferty JD, Gibson J, Shirali M *et al.* Genome-wide meta-analysis of depression identifies 102 independent variants and highlights the importance of the prefrontal brain regions. *Nat Neurosci* 2019; **22**: 343–352.](http://paperpile.com/b/1nD4ZF/IHPQI)

22 [Trubetskoy V, Pardiñas AF, Qi T, Panagiotaropoulou G, Awasthi S, Bigdeli TB *et al.* Mapping genomic loci implicates genes and synaptic biology in schizophrenia. *Nature* 2022; **604**: 502–508.](http://paperpile.com/b/1nD4ZF/z6H6N)

23 [Scott RA, Scott LJ, Mägi R, Marullo L, Gaulton KJ, Kaakinen M *et al.* An Expanded Genome-Wide Association Study of Type 2 Diabetes in Europeans. *Diabetes* 2017; **66**: 2888–2902.](http://paperpile.com/b/1nD4ZF/J0X3Y)

24 [Cox DR, Snell EJ. *Analysis of Binary Data, Second Edition*. CRC Press, 1989.](http://paperpile.com/b/1nD4ZF/K385Q)

25 [Nagelkerke NJD. A note on a general definition of the coefficient of determination. Biometrika. 1991; **78**: 691–692.](http://paperpile.com/b/1nD4ZF/2N30J)

26 [Stenbacka M, Jokinen J. Violent and non-violent methods of attempted and completed suicide in Swedish young men: the role of early risk factors. *BMC Psychiatry* 2015; **15**: 196.](http://paperpile.com/b/1nD4ZF/pZfzo)

27 [Kanehisa M, Goto S. KEGG: kyoto encyclopedia of genes and genomes. *Nucleic Acids Res* 2000; **28**: 27–30.](http://paperpile.com/b/1nD4ZF/2KeNl)

# Supplementary Figures and Legends


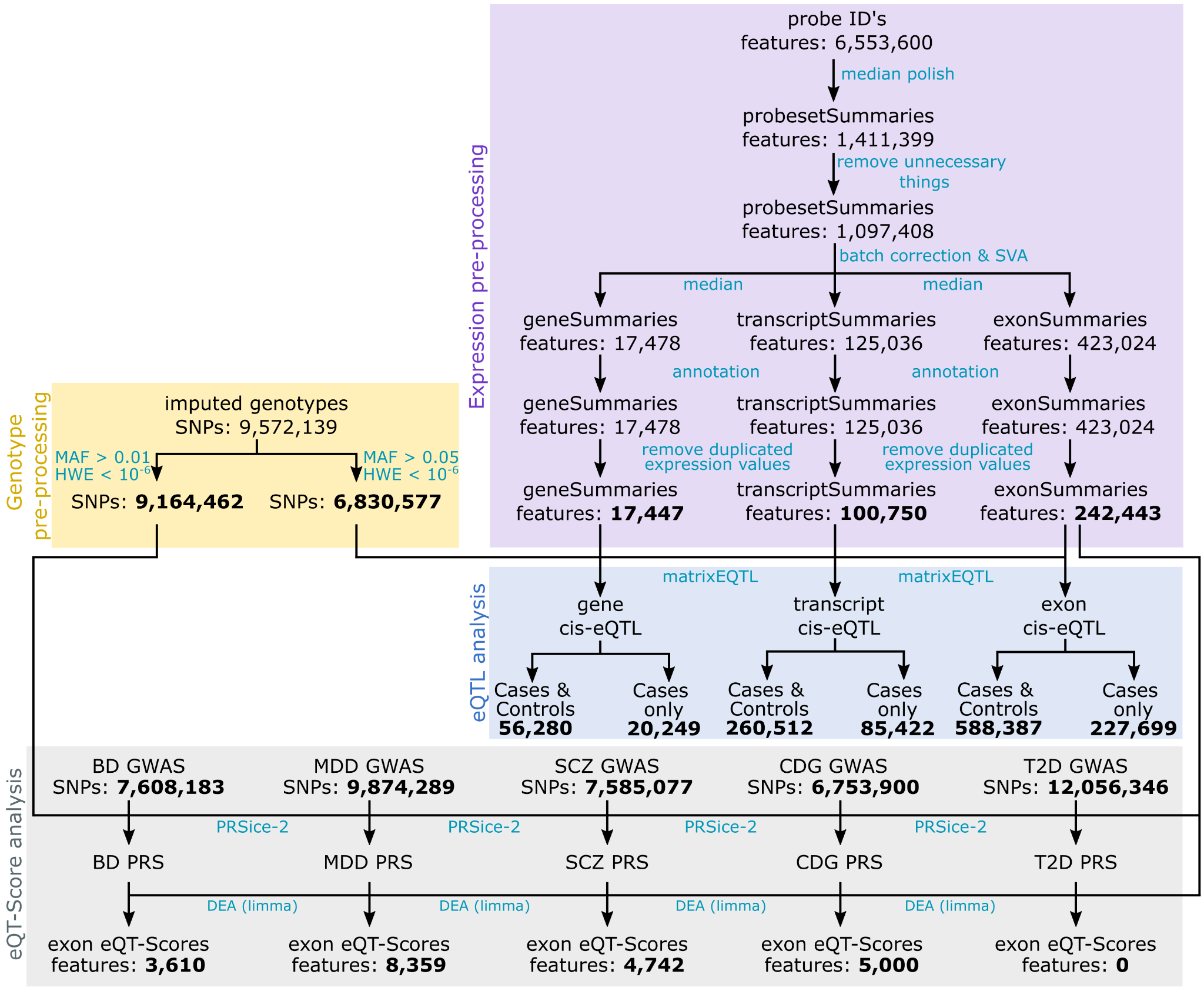
[**Figure S1:**](#sfigu_overview) **Overview of the microarray pre-processing steps.** The genotype pre-processing (yellow box) splits into two sections: 1) The genotypes are filtered for a minor allele frequency (MAF) > 0.01 and a Hardy-Weinberg equilibrium (HWE) < 10^-6^. Resulting SNPs were used for polygenic risk score (PRS) calculations and eQT-Score analysis (light gray box). 2) Genotypes were filtered for MAF > 0.05 and HWE < 10^-6^ for the eQTL analysis. The expression pre-processing (purple box) comprises several steps, including a self-made median summarization to gene-, transcript-, and exon-level summarization. Both genotypes and expressions were combined for the eQTL analysis (blue box). The turquoise texts next to the arrows show either the used method/tool or a short description of the task done at this step. Bold numbers display the number of resulting data values used for the following steps.

**
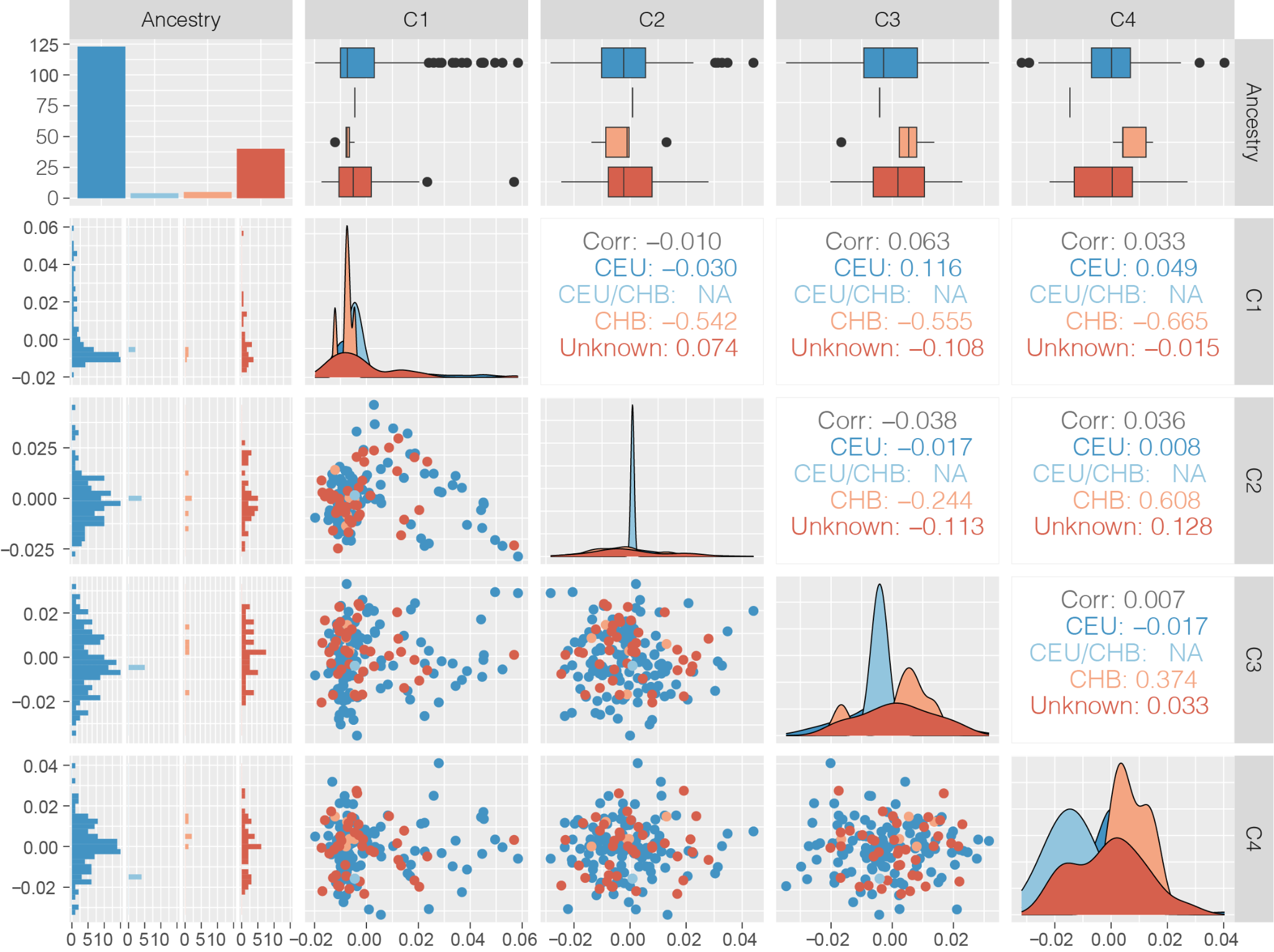
**

[**Figure S2:**](#sfigu_covariates) **Visual presentation of ancestry distributions.** MDS plot depicting known ancestry in relation to the first four genetic dimensions. The initial plot in the upper-left corner presents a bar plot illustrating known ancestry counts. Most are of Caucasian ancestry (n=123, CEU, blue), followed by some with no ancestry information (n=40, Unknown, red), a small proportion of Chinese ancestry (n=5, CHB, orange) and one that is both (n=1, CEU/CHB, light blue). Below, facet histograms and top-row boxplots showcase the data composition for each ancestry group. The remainder of the plot includes the Pearson correlation coefficient in the upper triangle, distributions along the diagonal, and PCA plots of distinct dimensions in the lower triangle. Notably, no discernible clusters were observed.


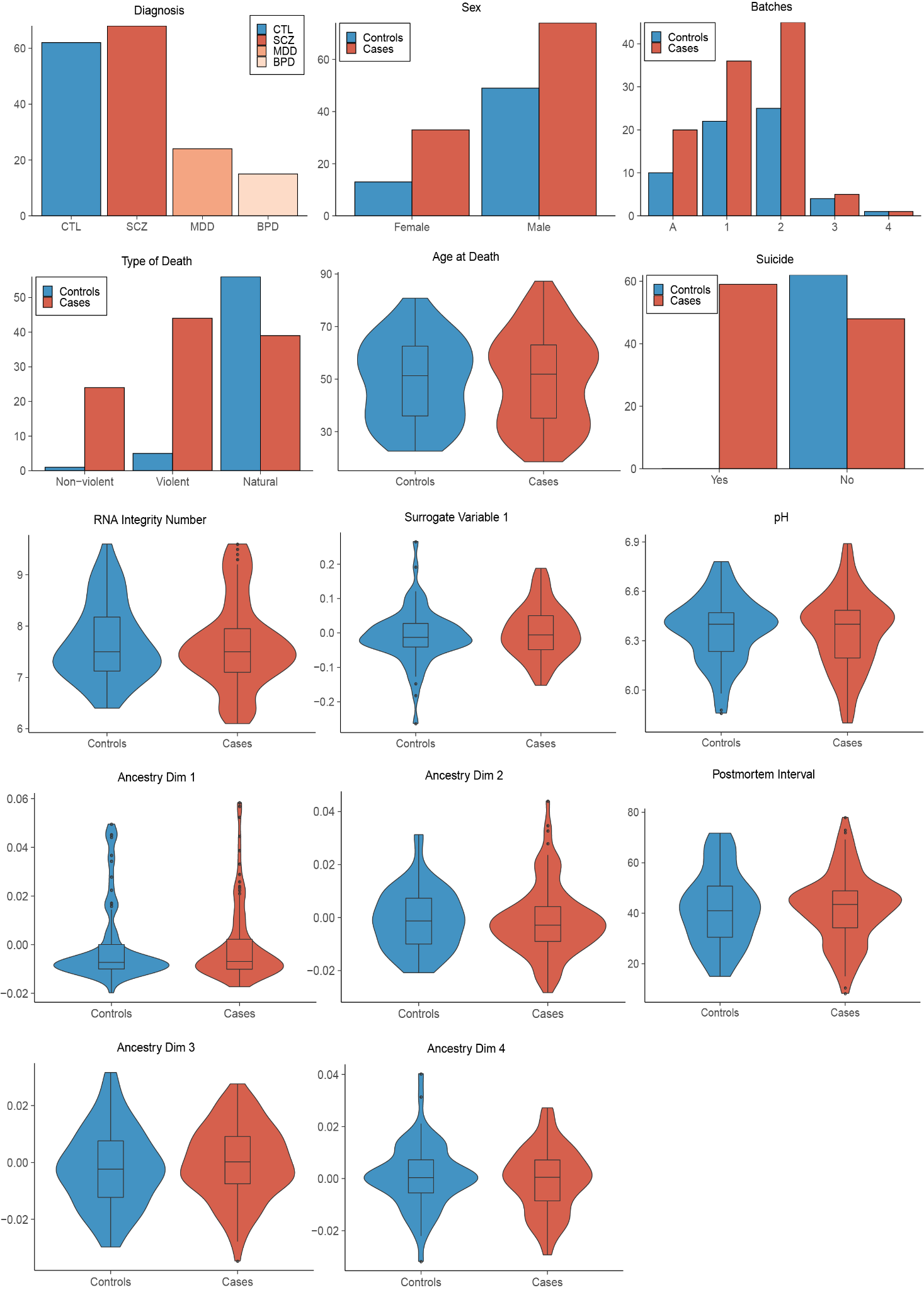


[**Figure S3:**](#sfigu_covariates) **Visual presentation of covariate distributions.** Distribution comparison between cases (in red) and controls (in blue) for diagnostic categories, microarray batches, and all covariates integrated into the linear model. Categorical data is depicted as barplots, while continuous data is represented as violin plots, each containing embedded boxplots.


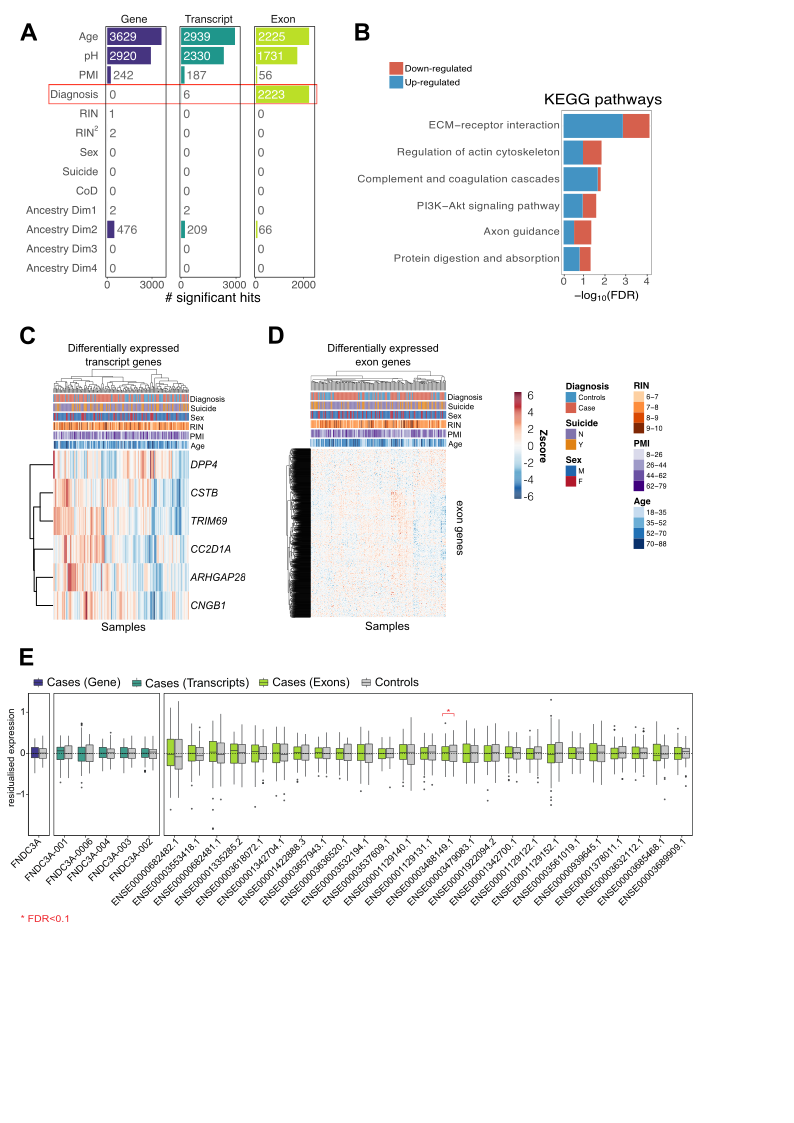


[**Figure S4:**](#sfigu_degenes) **Differential expression results.** A) Bar plots showing the differentially expressed gene hits (FDR < 0.1) for all model covariates on gene (orange), transcript (blue) and exon (green) level. The red box highlights the findings between cross-disorder cases and controls for diagnosis. B) Pathway enrichment of the 2,223 exon-level differentially expressed genes using FUMA with KEGG [^27^](https://paperpile.com/c/1nD4ZF/2KeNl), where disease and drug development pathways were excluded. Only significantly enriched (FDR < 0.05) KEGG pathways are shown coloured with the proportion of up- and down-regulated genes. Extracellular matrix (ECM)-receptor interaction and complement and coagulation cascade pathways contained 69% and 91% up-regulated genes, respectively. Conversely, 65% of the genes in the axon guidance pathway were down-regulated. C) Heatmaps of the six transcript-level differentially expressed genes and D) 2,223 exon-level differentially expressed genes. Heatmaps with default Euclidean distance and complete clustering method were used. E) Boxplots illustrating the effect of diagnosis on *FNDC3A* expression, separately for each level: full gene, five of its six transcripts and 25 of its 46 exons with available residualised expression values for cases (purple, turquoise, or light green) and control subjects (gray). The x-axis indicates expression residuals, and transcripts and exons are sorted by the difference in median expression from cases to controls. The asterisk indicates the only significantly differentially expressed exon (FDR<0.1).


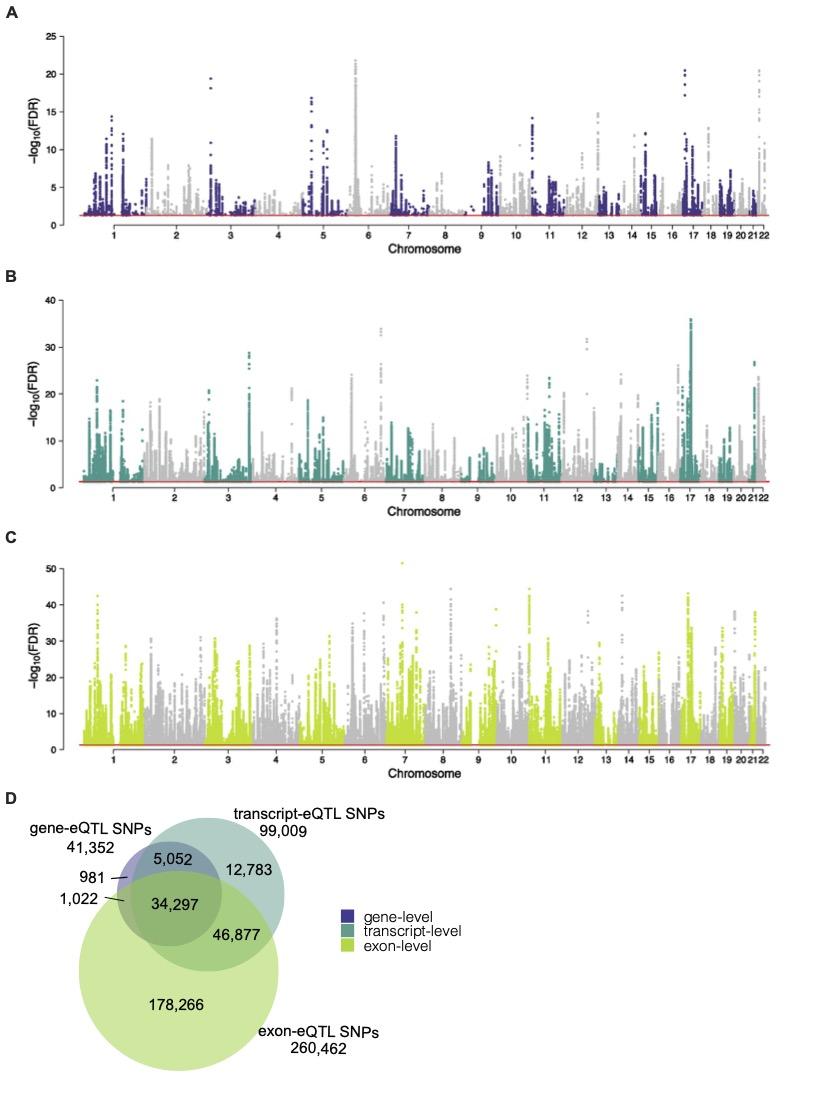


[**Figure S5:**](#sfigu_eQTLs) **eQTL results.** A-C) Manhattan plots of significant (FDR<0.05; indicated by horizontal red line) A) gene-, B) transcript- and C) exon-eQTL SNPs. D) Venn diagram illustrates the overlap of eQTL SNPs among all three levels, with 6% of gene-eQTL SNPs unique to this level and 65% exclusively detected at the exon-level.

# Supplementary Table Legends

[**Table S1**](#stabl_demographics)**:** Summary of the human postmortem brain samples and their demographic and clinical variability. Dataset 1: For continuous data the mean ± standard error and for categorical data the categories separated by dashes are given for control and case subjects as well as the different diagnosis types (schizophrenia, BD, MDD) of the cases. Age = years, postmortem interval (PMI) = hours (h), sex = males (M) and females (F), suicide = yes (Y) and no (N) and cause of death (CoD) = grouped into natural (N), non-violent (NV) and violent (V) death. Dim 1 - 4 = the first four dimensions of the ancestry information calculated with the sample genotypes. Dataset 2a: Given are the diagnosis, sex differentiated between male (M) and female (F), age in years, post-mortem interval (PMI) in hours (h), cause of death (CoD) and ethnicity

[**Table S2**](#stabl_degenes): i) Gene-, ii) transcript- and ii) exon-level differentially expressed genes for diagnosis. Note. Ensembl Gene ID = stable Ensembl gene identifier; Ensembl Transcript ID = stable Ensembl transcript identifier; Ensembl Exon ID = stable Ensembl exon identifier; Location = gene, transcript or exon location including chromosome, start and end position; Gene Symbol = HGNC or MGI-approved gene symbols; logFC = estimate of the log2-fold-change corresponding to the effect or contrast; Average Expression = average log2-expression over all samples; T-statistic = moderated t-statistic (log2FC divided by its standard error); P-value = raw p-value; FDR estimate = Benjamini-Hochberg false discovery rate adjusted p-value; Log-odds Ratio = log-odds that gene, transcript or exon is differentially expressed.

[**Table S3**](#stabl_geQTL): List of gene-level cis-eQTL results. Note. SNP = dbSNP rs ID; Ensembl Gene ID = stable Ensembl gene identifier; Gene Symbol = HGNC or MGI-approved gene symbols; Beta = effect size estimate; T-statistic = test statistic (t-test); P-value = raw p-value; FDR estimate = Benjamini-Hochberg false discovery rate adjusted p-value; SNP Location = SNP chromosome and base pair position; SNP_allele1 = reference allele of the SNP; SNP_allele2 = alternate allele of SNP; Gene Location = gene location given as chromosome, start and end position; Gene Strand = DNA strand where the gene is located; eQTL Type = information if eQTL is of type cis or trans.

[**Table S4**](#stabl_teQTL): List of transcript-level cis-eQTL results. Same column labels as [Table S3](#sta_geQTL) + Transcript ID = stable Ensembl transcript identifier; Transcript location = position of transcript with chromosome, start and end position.

[**Table S5**](#stabl_eeQTL): List of exon-level cis-eQTL results. Same column labels as [Table S4](#sta_teQTL) + Exon ID = stable Ensembl exon identifier; Exon Location = position of exon with chromosome, start and end position.

[**Table S6**](#stabl_annotation)**:** i) eSNP annotation to Variant Effect Predictor (VEP) categories. ii) eSNP annotation to ChromHMM DLPFC categories. iii) eSNP annotation to genome-wide association studies (GWAS) of different phenotypes. iv) eSNP annotation to CMC eQTL and isoQTL SNPs. v) eSNP annotation to GTEx DLPFC BA9 eQTL and sQTL SNPs.

[**Table S7**](#stabl_prs)**:** i) GWAS summary statistics overview used for the PRS calculation. ii) P-values, Nagelkerke and Cox & Snell R^2^ of PRSice-2 calculated PRS. Values are given for the p-value thresholds 1, 0.01, 5e-08 and the best PRSice-2 calculated threshold.

[**Table S8**](#stabl_eQTScore)**:** List of unique Ensembl Gene IDs and gene symbols from the BD, MDD and SCZ joint exon eQT-Score dataset.

[**Table S9**](#stabl_core)**:** List of unique Ensembl Gene IDs and gene symbols from the overlapping core set (n = 110) of exon-eQTL genes, genes from rare coding variants from SCHEMA consortium and the joint exon eQT-Score dataset.

**Table S10**: List of enriched KEGG pathways, including the name of the pathway, number of genes in each pathway, number of overlapping genes to the core gene set, calculated p-value, adjusted p-value and gene symbols of overlapping genes.
